# Scoping review of methods of monitoring acute changes in lower body neuromuscular function via force plates

**DOI:** 10.1371/journal.pone.0322820

**Published:** 2025-05-09

**Authors:** Andrew J. Badby, Nicholas J. Ripley, John J. McMahon, Peter D. Mundy, Paul Comfort

**Affiliations:** 1 Centre for Human Movement and Rehabilitation Science, School of Health and Society, University of Salford, Salford, United Kingdom; 2 Hawkin Dynamics, Inc., Westbrook, Maine, United States of America; 3 Strength and Power Research Group, School of Medical and Health Sciences, Edith Cowan University, Joondalup, Western Australia, Australia; University of Belgrade: Univerzitet u Beogradu, SERBIA

## Abstract

Force plates are amongst the most utilised technological apparatus for monitoring acute changes in neuromuscular function in sports. Practitioners apply monitoring strategies to manage neuromuscular fatigue and physical preparedness with valid, reliable, and sensitive measures. The aim of this scoping review was to identify, map, and describe the variety of monitoring procedures which have been previously applied in research (e.g., test and metric selection, data collection, study design, and data analysis procedures). Searches were completed by 24th June 2024. One thousand, nine hundred, and seventy-eight studies were identified across four databases (PubMed, EBSCO, Clarivate web of science, and Ovid). After removing duplicates, applying the inclusion criteria, and scouring the reference lists of remaining studies, a final total of thirty studies of within-group repeated measures design were used in this review. Major differences were identified across all aspects of studies methodologies, such as in subject demographics (e.g., sex, sport, and competitive level), data collection protocols (e.g., force plate hardware utilised, test and metric selection, verbal cues, and provision of information regarding testing surface, familiarisation and warm-up provided, the process of zeroing force plates between trials, and weighing of subjects during trials), and study design (e.g., reference physical activity investigated, time of season, testing timepoints, and training load determination). A general lack of reporting and uniformity in metric definitions, metric calculations, and phase terminology was identified across studies. For example, two separate calculations were reported for “peak force” across studies, as either “the maximum force achieved throughout the entirety of the trial”, or as “the maximum force achieved during the propulsion phase”. The latter calculation was also utilised for “peak concentric force” in a separate study. Thus, an accurate comparison of results across studies (e.g., via meta-analysis) and forming any generalized conclusions about the application of specific tests and metrics for monitoring acute changes in neuromuscular function using force plates was premature at this time. The information presented in this review will contribute towards forming a rationale for the data collection, study design, and data analysis protocols for future research on monitoring acute changes in neuromuscular function using force plates.

## Introduction

Practical and accurate technological solutions which boast validated hardware [1] and software [2] against criterion methods have contributed to an increased popularity of physical evaluation practices (e.g., physical profiling and fatigue monitoring) in sports, with many strength and conditioning coaches testing their athletes daily [3]. Taylor et al. [4] conducted a survey to identify contemporary physical evaluation practices by sports science practitioners in a variety of sports (e.g., rugby union, soccer, Australian rules football, swimming, track & field, etc.). Of 100 invites, 55% of practitioners responded, and 91% of these reported the use of some form of monitoring or evaluation of neuromuscular function (NMF) in response to training or competition. The aims of these assessments were to prevent overtraining (22%), reduce the likelihood of non-contact soft-tissue injury (29%), determine the effectiveness of training programs (27%), and ensure the maintenance of performance throughout periods with high fixture congestion (22%) [4]. The methodologies of physical evaluation practices also varied, but the most utilised were self-reporting (e.g., daily wellness) questionnaires (84%), and tests which provided objective measures of NMF (61%) [4], performed on a monthly (33%), weekly (30%), or daily (6%) basis [4]. Vertical jump (VJ) tests were most commonly used (54%), where the countermovement jump (CMJ) test was utilised most frequently (N = 11), whilst the broad jump and squat jump (SJ) tests featured only once [4]. Objective measures are the most effective means of monitoring the magnitude and time course of acute changes in NMF [5]. The continuous monitoring of NMF on a weekly and/or daily basis can be used to determine neuromuscular fatigue and physical preparedness [6]. Additionally, if performed over the period of a season, continuous monitoring can be used to determine chronic developments in fitness [7,8]. Continuous monitoring may be favourable in addition to the “traditional approach” of performing large scale fitness testing batteries 3–4 times per season (i.e., adopting a “combined approach”) given that more frequent, smaller scale, testing sessions consisting of specific key measures will provide more data points within a given period, potentially resulting in more informative data trends in reports [7,8], and providing a better understanding of individual injury risk [9,10]. Force plates are amongst the most utilised technological apparatus for collecting objective measures of NMF in sports [4].

### Data collection considerations

For force plate data to be beneficial [11], tests and metrics must (a) measure what they are supposed to (demonstrate validity) [1], (b) relate to physical performance in competition (be meaningful) [12], and (c) be sensitive enough to detect change (possess high reliability and low measurement error) [11]. Additionally, specific to elite sports settings, tests are only useful for continuous monitoring if they are feasible in a practical setting. To ensure testing feasibility, examples of required characteristics include (d) quick and easy to perform (time-efficient) and (e) mechanically and metabolically non-fatiguing in nature (low physical demand) [13–16]. To add to this, coaches also value (f) the ability to immediately and appropriately analyse, interpret, and act upon testing data, which is a quality that is available via some force plate systems with integrated proprietary software [2]. Regarding the meaningfulness of measures, actions in sports typically utilise the stretch shortening cycle (SSC), where a muscle is first actively lengthened (eccentric action) followed by an immediate shortening (concentric action) [17,18]. Consequently, lower-body NMF is commonly assessed via tests which utilise the SSC, such as VJ tests [4]. Common examples of VJ tests include the bilateral CMJ [19,20], unilateral CMJ [20,21], drop jump (DJ) [22,23], and other variations of rebound jump (RJ) [24,25] tests. With regards to the sensitivity of measures to detect change [11], more technique-intensive tests exhibit greater data variability, and require more practice to produce consistency and reliability [11]. If a test requires a technique in which the athlete has not developed consistency, the test itself may fail to provide consistent results [26,27].

In a recent scoping review, Guthrie et al. [12] examined the practices used for evaluating changes in physical preparedness longitudinally (e.g., pre- to post- training programme) within the football codes (i.e., rugby, soccer, American football, and Australian rules football). Guthrie et al. [12] identified the most frequently used test of NMF was the CMJ and suggested that this seems likely because of the test’s ease, time-efficiency, and lack of resources required, leading to high compliance seen in the practical setting. Concentric-only (e.g., the SJ) [28] and isometric (e.g., the isometric mid-thigh pull [IMTP]) [29] tests were reported as utilised less frequently [12]. A scoping review of methods used for the continuous monitoring of acute changes in NMF, and outside of the football codes, is yet to be performed. Athletes have a limited time to produce ground reaction force (GRF) in most competitive sports, so it is important to evaluate NMF using metrics which represent both the outcome (e.g., take-off velocity [TOV] and jump height [JH]) and the strategy (e.g., time to take-off, countermovement depth, ground contact time [GCT], etc.) performed [30]. Gathercole et al. [31] also recommended utilising both outcome (e.g., JH) and strategy (e.g., time to take-off) metrics independently, given the explained time constraints in competitive sports, and because neuromuscular fatigue can manifest as an alteration in movement strategy highlighted by changes in temporal characteristics, without a change in outcome [32]. It is also important to consider kinetic measures as their generation throughout braking and propulsion is what dictates the movement strategy performed, and ultimately influences overall task outcome (i.e., TOV and JH) [33,34]. Further, ratio measures such as the reactive strength index (RSI) in RJ tests and modified reaction strength index (mRSI) in the CMJ test have also been proposed in previous research as they incorporate factors relating to both the outcome (i.e., JH) and strategy (i.e., GCT and time to take-off, respectively) performed [25,35–37].

Merrigan et al. [14] applied a random forest regression model to identify the metrics that were most influential to CMJ outcome (i.e., JH), reporting concentric (i.e., propulsion) duration, countermovement depth, and eccentric-to-concentric mean force ratio as the most significant predictors of JH, with a combined 91.7% explained variance. Based on the impulse-momentum theorem, net propulsive impulse (propulsive force multiplied by propulsive phase time) relative to mass determines the vertical TOV of the centre of mass (COM) [30], which determines JH [38]. Consequently, it is not surprising that Merrigan et al. [14] proposed metrics relating to GRF production (i.e., eccentric-to-concentric mean force ratio) and strategy (i.e., concentric [also referred to as propulsion] duration and countermovement depth) as the most meaningful for evaluating NMF [14]. However, there is a reported mismatch between these proposed CMJ metrics and those most frequently used for evaluating changes in physical preparedness longitudinally, which Guthrie et al. [12] reported were JH (N = 6), flight time (FT) (N = 2), flight time contraction time ratio (FT:CT; N = 2), peak force (N = 1), peak power (N = 1), and relative peak power (N = 1).

When monitoring acute changes in NMF, the outcome of a VJ task is determined by relative net propulsive impulse [30,39,40]. However, significant (*p* < 0.05) reductions in body mass have been reported following both recreational (1.6 ± 0.2%) and professional (1.9 ± 0.2%) soccer matches, which would render outcome measures quite limited for detecting changes in NMF if body mass changes significantly across testing timepoints (e.g., pre- to post-match) [41]. This also extends to ratio metrics such as FT:CT and RSI, as outcome measures feature within their equations [42]. Additionally, mean and peak values (e.g., mean and peak force and power) are affected by changes in strategy, where an acute change to a shallower (i.e., “stiffer”) strategy can elicit greater forces over less time, and likely result in a reduced net propulsive impulse [43]. Thus, an alteration in strategy would likely alter peak force and make it hard to determine any potential reductions in NMF that may have been detected when using this metric had the strategy remained the same. For example, researchers have reported large increases in time to take-off (effect size [ES] = 1.90) and eccentric time (ES = 1.91), along with decreases in absolute peak force (ES = 2.15) and eccentric-to-concentric mean power ratio (ES = 2.02), in CMJs at 30 minutes post-exercise (high intensity, interval style, stair climb protocol), but with a small increase in JH (ES = 0.47) [32]. This example strengthens the consideration to monitor more than outcome metrics as a small increase in outcome (i.e., JH) was observed (which could be deemed a positive change) but occurred due to changes in the performed strategy (e.g., increases braking and propulsive phase time and time to take-off), with a reduction in peak force [31,32,44]. To the author’s knowledge, the commonality of use of force plate derived outcome, strategy, kinetic, and ratio metrics for monitoring acute changes in NMF is yet to be determined.

Regarding the sensitivity of measures to detect change [11], to enable force-time data collected at different time points to be reliably compared (i.e., in the monitoring process), data collection procedures must consist of the same equipment, tests, and testers (to limit intra-rater variability), and be performed in the same environment (e.g., consistent flooring) [11]. Additionally, consistency in the zeroing of force plates between trials, cueing of technique (e.g., ‘jump as fast and high as possible’), trial exclusion criteria (e.g., arm swing [AS] or tucking of the legs during a VJ [the latter does not matter if calculating JH from TOV]), and application of the pre-determined study design protocol (i.e., how many trials, over how many sessions?) in every testing session (i.e., over multiple time-points) is essential if utilising the data to monitor changes in NMF to inform training prescription [12]. It is also a requirement that testing sessions are performed with identical data collection procedures so that any difference between two sets of scores is limited to factors such as intra-subject (i.e., same subject) biological variability [11]. To the author’s knowledge, the commonality of force plate data collection and testing standardisation procedures used for monitoring acute changes in NMF is yet to be determined. Consistency in the utilised and reported data collection and testing standardisation procedures in literature is essential to enable future comparisons of data (e.g., in meta-analyses), and would contribute towards the aim of promoting an accurate and consistent implementation of force plate testing in practice [12,26].

## Objectives

A variety of options are available to sports science practitioners to monitor acute changes in NMF using force plates, thus, researchers use different approaches regarding force plate test and metric selection [12]. The primary aim of this scoping review was to identify, map, and describe which practices exist in the context of monitoring acute (<1 week pre- to post-physical stimulus) changes in NMF using force plates. In a qualitative manner, the review will describe the similarities, differences, and gaps in the body of evidence retrieved regarding the specific force plate data collection, study design, and data analysis procedures employed. This knowledge will contribute towards forming a rationale for the data collection, study design, and data analysis protocols in future research on monitoring acute changes in NMF using force plates. To the author’s knowledge, no review currently exists which serves this purpose.

## Methods

### Design

This scoping review followed the latest methodological guidance of the Preferred Reporting Items for Systematic Reviews and Meta-Analyses (PRISMA) extension for Scoping Reviews (refer to S1 Table) [45], with consideration for recommendations in research relating to scoping review protocols [45–48]. The objectives of this review were also developed considering the Population, Concept, Context (PCC) framework, as advised in a recent best practice reporting items protocol guide for the development of scoping reviews [46]. To allow for a broad identification of monitoring applications, the *population* specification was athletes of any sport (individual or team), participants whose occupation involved physical demand (i.e., the public services and military) in which the evaluation of NMF has been reported [23,49], university students engaged in a sports team or course (e.g., sports science), and recreational athletes were also included. The *concept* of the research was to determine force plate methods previously used to evaluate NMF in the *context* of monitoring acute changes in daily or weekly monitoring or in response to a physical stimulus (e.g., a training session or competitive match). Based on this, acute, within-group, repeated measures design research was expected upon retrieval. A review protocol was not pre-registered for this review.

### Literature search

A Boolean/phrase search mode was applied using the following key words: “force plat*” AND “athlete” OR “player” OR “game” OR “match” OR “competition” OR “season” OR “sport” AND “monitoring” OR “testing” OR “evaluation” OR “assessment” OR “profiling” OR “benchmarking” OR “programming” OR “supercompensation”. Parentheses were utilised for grouping terms together (i.e., those combined with “OR”), and thus separating the groups of terms (i.e., those separated with “AND”) during the searches. The keywords were inputted using this format into the following four databases: PubMed, EBSCO, Clarivate web of science, and Ovid. Filters were applied to all databases such as the key words were present in the topic (i.e., title, abstract, or key words) of studies written in the English language and presented in peer-reviewed academic journal articles. No restrictions were placed upon the age or sex of subjects. The search timeframe was not date restricted and was completed by 24th June 2024.

### Inclusion and exclusion criteria

**Fig 1:**
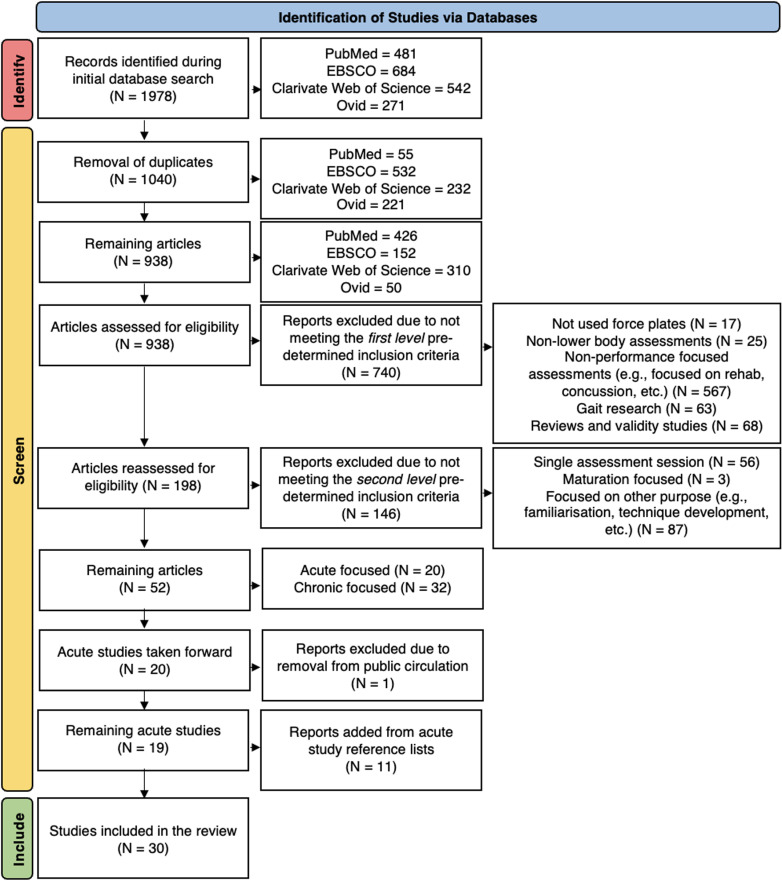
Flowchart illustrating the study selection process.

All duplicates were removed initially with the remaining studies then being screened utilising the following inclusion criteria. The inclusion criteria were applied in three stages (Fig 1).

Initially, a *first level* criterion was applied where research articles were deemed eligible provided that the study [1] was written in the English language, [2] reported the use of force-plates, [3] reported the purpose of testing was to assess *lower-body* NMF, [4] reported the purpose of testing was to identify physical capacity (fitness) or an acute or chronic response to a stimulus (fatigue or fitness, respectively), and [5] reported testing a physically abled population. Articles were excluded if the study [1] was not written in the English language, [2] did not clearly report the use of force-plates, [3] did not report the use of *lower-body* assessments or reported only *upper-body* assessments, [4] reported a purpose of testing other than to identify to identify physical capacity (fitness) or a response to a stimulus (fatigue), such as for rehabilitation progression, concussion recovery, balance or gait, and [5] reported testing with a population with a physical disability. All reviews and validation studies (e.g., force-plates vs linear position transducers) were excluded.

A *second level* inclusion criterion was then applied, where research articles were deemed eligible provided that the study [1] reported more than one testing session was performed, and [2] reported a focus on monitoring *changes* in NMF. Studies were excluded if the study [1] reported the use of only a single testing session, or [2] reported a focus other than monitoring *changes* in NMF, such as changes in biological maturation or developments in a specific sports task (e.g., golf drive kinetics).

A *third level* inclusion criterion was then applied, where the remaining studies were categorised as focused on acute (<1 week between physical activity and re-test) or chronic (>1 week between physical activity and re-test) changes in NMF. The remaining acute studies (N = 11) were taken forward for review, and as performed in recent similar research [12], the reference lists of these studies were then examined for additional studies that could be included in the review (Fig 1).

### Analysis and interpretation of results

After the inclusion criteria was applied the remaining studies were evaluated and reported. As performed in recent similar research [12], the information extracted from studies was primarily qualitative, which included demographics (e.g., sport, sex, age, height, mass, competitive level, experience), descriptive data collection information (e.g., number of tests and metrics used, frequency of testing, timelines of testing, time of season, verbal cues, surface, familiarisation, warm-up, zeroing of force plates, and weighing of subjects), activity information (e.g., activity performed and measures of quantifying training load [TL]), force-plate hardware used, and data analysis procedures (i.e., calculations including differences in metric definitions). Quantitative information was extracted from these studies only to identify how many studies provided sufficient results information (i.e., mean and standard deviation [SD]) at each time point. This quantitative data served to allow for subsequent discussions of information regarding data collection, metric selection, and data analysis procedures.

## Results

### Search results

Refer to Fig 1 for a flowchart illustrating the study selection process. One thousand, nine hundred, and seventy-eight studies were identified within the four databases, with one thousand and forty studies being duplicates and therefore removed prior to the application of the inclusion criteria (Fig 1). Nine hundred and thirty-eight studies were assessed for eligibility using the inclusion criteria, identifying eight hundred and eighty-six studies as ineligible (Fig 1). Of the remaining fifty-two articles, twenty were deemed as focused on ‘acute’ changes and thirty-two on ‘chronic’ changes in NMF. For this review, the acute studies were taken forward for further inspection, as a recent similar scoping review has already been performed with a longitudinal focus [12]. Upon further inspection, one study was identified as removed from circulation therefore the remaining acute studies included in this review was nineteen. After examining the reference lists of the remaining studies, an additional eleven studies were identified as appropriate to use in this review. Therefore, the remaining total of studies used in this review is thirty [21,31,32,44,50–75]. Studies are all of within-group repeated measures design (Fig 1).

### Summary of demographics

From the 30 articles assessed in this study, 466 participants were utilised resulting in an average sample of 15.5 ± 7.2 participants per study (Table 1). The average age, height, and body mass of participants in these studies was 22.8 ± 4.2 years, 182.9 ± 8.4 cm, and 82.4 ± 14.2 kg, respectively (Table 1). Regarding the demographics of participants, 26 studies included male (N = 381) participants [21,31,32,44,51–55,57,58,60–70,72–75], 5 studies included female (N = 53) participants [32,50,51,71,72], and 2 studies did not specify the participants’ sex (N = 32) [56,59] (Table 1). Consequently, male and female participants made up 81.76% and 11.37% of the total population of all studies, respectively, and 6.86% were not specified (Table 1).

**Table 1:** Summary of demographics.

Author(s)	Occupation/ Sport	Sample	Groupings	Sex	Age (years)	Height (cm)	Body Mass (kg)	Competitive Level	Experience
Bedo et al. [50]	Handball	20	Nil	Female	21.9 ± 3.4	176 ± 7	63.5 ± 9.1	Nil	7.2 ± 3.2 Playing Years
Boullosa et al. [51]	Runners and Triathletes	22	16 = Runners 6 = Triathletes	8 Runners = Male 8 Runners = Female 6 Triathletes = Male	Male Runners: 24 ± 4.3 Female Runners: 22.5 ± 5.5 Male Triathletes: 28.5 ± 6.2	Male Runners: 179 ± 8 Female Runners: 165 ± 6 Male Triathletes: 175 ± 5	Male runners: 68.4 ± 7.5 Female runners: 53.9 ± 3.8 Male triathletes: 67.2 ± 4.1	Mixed from “Regional” to “Elite”	Nil
Lonergan et al. [52]	Rugby Union	14	7 = < 24 Months Post ACL Reconstruction 7 = Non-injured	Male	Injured: 23 ± 3 Non-injured: 26 ± 3	Injured: 186 ± 4 Non-injured: 184 ± 7	Injured: 104 ± 7 Non-injured: 95 ± 7	Mixed with English Premiership, Championship, and National League 1	> 10 Playing Years > 2 Weeks Experience to Testing Task
Lupo et al. [53]	Rugby Union	9	6 = Backs 3 = Forwards	Male	Backs: 21 ± 1 Forwards: 20 ± 2	Backs: 182 ± 6 Forwards: 188 ± 5	Backs: 86 ± 7.4 Forwards: 97.2 ± 6.2	Italian Serie A	> 8 Playing Years
Scanlan et al. [54]	Basketball	10	Nil	Male	16.6 ± 1.1	182.4 ± 4.3	68.3 ± 10.2	Junior Level	Nil
McLellan et al. [55]	Rugby League	15	8 = Forwards 7 = Backs	Male	24.2 ± 7.3	188 ± 20.1	94.6 ± 26.8	“Elite” National Rugby League	Nil
Thorlund et al. [60]	Handball	10	Nil	Male	22.8 ± 1.5	188.4 ± 2.7	91.7 ± 3.0	“Elite” Danish National League	Nil
Gathercole et al. [31]	“Team Sports”	11	N/A	Male	23.8 ± 3.9	182 ± 6	80.3 ± 6.6	College Level	Nil
Kennedy et al. [44]	Rugby Union	9	Nil	Male	19.0 ± 1.5	188.3 ± 1.5	95.0 ± 10.5	“Elite” Academy Level	Nil
McLean et al. [61]	Rugby League	12	Nil	Male	24.3 ± 3.6	184.7 ± 6.1	101.9 ± 8.4	South Sydney Rabbitohs National Rugby League Team	69 ± 65 National Rugby League Matches
Roe et al. [62]	Rugby Union	14	Nil	Male	17.4 ± 0.8	182.7 ± 7.6	86.2 ± 11.6	Professional Rugby Union Academy	Nil
Yu et al. [63]	Mixed Recreational Sports University Students	15	N/A	Male	23.93 ± 0.80	176.70 ± 2.75	73.93 ± 4.76	N/A	Nil
Gathercole et al. [32]	Snowboard Cross	7	N/A	4 = Male 3 = Female	Male: 26.5 ± 5.8 Female: 26 ± 6.1	Male: 183.4 ± 3.8 Female: 165.7 ± 4.4	Male: 86.2 ± 3.4 Female: 64.4	“Olympic-caliber”	Nil
Oliver et al. [64]	Soccer	10	Nil	Male	15.8 ± 0.4	173 ± 6	59.8 ± 9.7	Amateur Youth Soccer Clubs	Nil
Clarke et al. [65]	Canadian Football	15	Nil	Male	21.8 ± 1.6	187.2 ± 5.2	97.6 ± 14.7	University of Saskatchewan	Nil
West et al. [66]	Rugby Union	14	Nil	Male	24.9 ± 4.4	185 ± 1	105.2 ± 12.3	Full-time Professional Celtic League and European Cup	Nil
Merrigan et al. [56]	Diplomatic Security Deployment Special Agents	13	N/A	Nil	37 ± 5	202.11 ± 28.71	71.67 ± 3.81	N/A	> 2 Years Consistent Physical Training
Horita et al. [67]	Mixed Recreational Sports	10	N/A	Male	28 ± 4	180 ± 6	78 ± 9	N/A	Nil
McCall et al. [58]	Soccer	29	23/29 Reliability Assessment 11/29 Sensitivity Assessment	Male	19.6 ± 3.5	181 ± 6	74.3 ± 7.4	French League 1 & Champions League	Nil
Travis et al. [59]	Powerlifting	19	Assigned to +3 Days (N = 10) or + 5 Days (N = 9) Re-test	Nil	23.8 ± 4.1	174.2 ± 7.3	90.8 ± 20.7	Nil	Nil
Bromley et al. [21]	Soccer	14	Nil	Male	17.6 ± 0.5	177 ± 8	63.2 ± 6.7	Academy Category 3	> 2 Years Soccer and Resistance Training Experience
Troester et al. [57]	Rugby Union	27	10 Backs 17 Forwards	Male	26 ± 3	189 ± 6	106 ± 14	Full-time professional	46 ± 22 Super Rugby League matches
Yoshida et al. [68]	Basketball	11	5 Guards 6 Forwards	Male	19.9 ± 1	187.6 ± 13.7	88.4 ± 12.2	Collegiate level (NAIA)	Nil
Cabarkapa et al. [69]	Basketball	17	Nil	Male	24.7 ± 6.8	194.7 ± 10.6	92.6 ± 9.9	First- and Second-tier European Leagues	Nil
Cabarkapa et al. [70]	Volleyball	10	Nil	Male	21.4 ± 2.2	197.3 ± 7.0	87.1 ± 7.3	Professional European SuperLeague	11.3 ± 2.4 Years Playing Experience
Donahue et al. [71]	Volleyball	11	Nil	Female	19.77 ± 1.09	178.56 ± 7.81	72.42 ± 7.81	NCAA Division 1	Nil
Janicijevic et al. [72]	Mixed Recreational Sports University Students	38	N/A	Male = 27 Female = 11	Male: 22.6 ± 3.7 Female: 21.9 ± 2.8	Male: 181 ± 5 Female: 166 ± 9	Male: 74.7 ± 7.2 Female: 57.3 ± 7.7	N/A	Nil
Spencer et al. [73]	Soccer	14	6 Defenders 5 Midfielders 3 Strikers	Male	26.6 ± 4.4	181 ± 5	79.8 ± 6.6	English National League	Tier 3 (i.e., highly trained/national-level)
Tazji et al. [74]	Basketball	30	Nil	Male	20.90 ± 1.49	163.2 ± 5.04	60.30 ± 3.10	Nil	Minimum 3 Years Playing Experience
Philipp et al. [75]	Basketball	16	Nil	Male	18–25	195.6 ± 10.4	93.2 ± 11.3	NCAA Division 1	> 4 Months Experience Performing Prescribed Tests

cm, centimetres; kg, kilograms; Nil, information not provided; N/A, not applicable; ACL, anterior cruciate ligament; NAIA, National Association of Intercollegiate Athletics; NCAA, National Collegiate Athletic Association.

Of these 30 studies, 26 studies specified the use of a population engaged in competitive sports [21,31,32,44,50–55,57–62,64–66,68–71,73–75], 3 studies with mixed recreational sports athletes [63,67,72], and 1 study with military personnel [56] (Table 1). Of the competitive sports populations, 23 studies recruited team sports athletes (e.g., soccer) [21,31,44,50,52–55,57,58,60–62,64–66,68–71,73–75], and 3 studies recruited individual sports athletes (i.e., triathlon, snowboard cross, and powerlifting athletes) [32,51,59] (Table 1). The most frequent competitive sports were Rugby Union (N = 6; [44,52,53,57,62,66]), Basketball (N = 5; [54,68,69,74,75]), Soccer (N = 4; [21,58,64,73]), Volleyball (N = 2; [70,71]), Rugby League (N = 2; [55,61]), and Handball (N = 2; [50,60]), all of which are team sports (Table 1).

Of the 26 studies which specified the use of a population engaged in competitive sports, 14 studies defined their sample as “elite” or full-time professional athletes (i.e., “senior” level) [32,44,51–53,55,57,58,60,61,66,69,70,73], 10 studies used participants which were considered “college”, “youth”, or “academy” level [21,31,44,54,62,64,65,68,71,75], and 2 studies did not specify [50,74] (Table 1). Finally, 11 studies reported training or playing experience [21,50,52,53,56,57,61,70,73–75], and 19 studies did not [31,32,44,51,54,55,58–60,62–69,71,72] (Table 1).

### Summary of data collection protocols

From the 30 articles assessed in this study, the tests utilised were the CMJ (N = 23; [31,32,44,50–55,60–66,68–73,75]), DJ (N = 3; [56,64,67]), SJ (N = 1; [64]), isometric posterior lower-limb muscle test (N = 1; [58]), isometric squat test (N = 1; [59]), unilateral CMJ (N = 3; [21,52,72]), unilateral drop landing (N = 1; [57]), bilateral hopping (N = 1; [74]), and the 10/5 RJ test (N = 1; [75]) (Table 2). Of the studies involving competitive sports populations (N = 26), most of the testing took place during the in-season period (N = 17; [21,32,44,51–53,55,57,58,60–62,64,66,69,70,73]) in comparison to the pre-season (N = 2; [65,75]) and immediately post-season (N = 1; [51]) periods, whilst some did not specify when within the season the testing was done (N = 6; [31,50,54,68,71,74]) (Table 2). Regarding the standardisation of testing procedures, 24 studies defined the use of verbal cueing for testing [21,44,51–58,60–62,64–66,68–75], whilst 6 did not [31,32,50,59,63,67]. The most common trial instructions used included “hands-on-hips” (N = 15; [21,44,53,54,60–62,64–66,69,70,73–75]), “jump as high as possible” (N = 12; [44,51,54,55,60–62,64–66,71,72]), and with a “self-selected countermovement depth” (N = 14; [21,44,51–55,61,62,64–66,71,73]) (Table 2).

**Table 2:** Summary of reported data collection protocols.

Author(s)	Test	Time of Season	Verbal Cues	Surface Used?	Familiarisation?	Warm-Up?
Bedo et al. [50]	CMJ	Nil	Nil	Nil	Nil	Nil
Boullosa et al. [51]	CMJ	Runners: Immediately Post-season Triathletes: Mid- to End-season	Maximal effort CMJ Jump as high as possible Countermovement depth freely chosen	Nil	A single laboratory session for familiarisation to anthropometric and CMJ performance tests between 48 h and 1 week before the testing session.	Nil
Lonergan et al. [52]	Bilateral CMJ Unilateral CMJ	In-season	Hands on hips Jump as fast and high as possible Self-selected countermovement depth	Nil	Nil	Nil
Lupo et al. [53]	CMJ	In-season	Hands on hips Jump with freely chosen strategy	Nil	Nil	Nil
Scanlan et al. [54]	CMJ	Nil	Hands on hips Jump as high as possible Self-regulated countermovement depth	Nil	Separate session of CMJ familiarisation in a biomechanics laboratory	15 minutes of jogging, whole-body dynamic stretches, and repeated high-intensity bouts
McLellan et al. [55]	CMJ	In-season	Arm swing utilised Jump as high as possible Arm swing technique and countermovement depth was self-determined	Nil	A single sub-maximal CMJ trial prior to data collection	10 minutes of self- paced stationary cycling followed by 5 minutes of prescribed dynamic stretching
Thorlund et al. [60]	CMJ	In-season	Hands on hips Make a fast downward movement to about 90 degrees knee flexion immediately followed by a fast upward movement Jump as high as possible	Nil	Subjects visited the laboratory on a separate day for familiarization to the test procedures	10 minutes on a Monark ergometer cycle (90 RPM, 2 kg resistance, 180 W)
Gathercole et al. [31]	CMJ	Nil	Nil	Nil	Warm-up and CMJ practice with an emphasis on the speed of jump until demonstration of consistent CMJ technique, performed 7-days before testing	20 minutes of light jogging, dynamic stretching, and 10- and 20-metre sprints
Kennedy et al. [44]	CMJ	In-season	Hands on hips Jump as high as possible Countermovement depth was self-determined	Nil	Nil	10 minutes of jogging, dynamic stretching, and 5 sub-maximal CMJs
McLean et al. [61]	CMJ	In-season	Hands on hip Jump as high as possible Self-selected countermovement depth	Nil	Familiarised with protocols during regular training sessions	5 minute dynamic warm-up
Roe et al. [62]	CMJ	In-season	Hands on hips Jump as a high as possible Countermovement depth was self-determined	Nil	Nil	2 minutes of dynamic stretching
Yu et al. [63]	CMJ	N/A	Nil	Nil	Nil	Nil
Gathercole et al. [32]	CMJ	In-season	Nil	Nil	Familiarised with protocols during regular training sessions	15 minutes of light cycling, dynamic stretching, and 10- and 20-metre sprints
Oliver et al. [64]	CMJ DJ SJ	In-season	Hands on hips for all jumps CMJ: Jump as high as possible Countermovement depth was self-selected SJ: Jump as high as possible from 90 deg knee flexion angle and without a countermovement DJ: Drop from a height of 0.35 m and then jump as high as you can as soon as possible after landing	Nil	Practice testing session 1 week before testing	Nil
Clarke et al. [65]	CMJ	Pre-season	Hands on hips Jump as high as possible No directions were given regarding movement speed or countermovement depth	Nil	2-5 minute familiarisation prior to testing	12 minutes of jogging followed by 8 min of dynamic movements
West et al. [66]	CMJ	In-season	Hands on hips Self-selected countermovement depth “ Explode” upwards and jump as high as possible	Nil	Familiarised with protocols during regular training sessions	5 minutes of jogging, dynamic stretching, and 3 maximal effort CMJs
Merrigan et al. [56]	DJ	N/A	Step off (not walk or jump off) the box and immediately perform a maximal effort CMJ with little ground contact	Nil	Familiarised with protocols during regular training sessions	5 minutes of cycling and 5 minutes of dynamic stretching
Horita et al. [67]	DJ	N/A	Nil	Nil	Nil	Nil
McCall et al. [58]	Isometric Posterior Lower-limb Muscle Test	In-season	The player pushed their heel into the force platform as hard as possible without lifting their buttocks, hands or head off the mat Verbal encouragement was given	Nil	2 familiarisation sessions 1 week before testing	7 minutes pedalling at 90 W followed by 3 min at 120 W on a cycle ergometer
Travis et al. [59]	Isometric Squat	N/A	Nil	Nil	Nil	Nil
Bromley et al. [21]	Unilateral CMJ	In-season	Hands on hips Self-selected countermovement depth Maximal effort to jump as fast and as high as possible The non-jumping limb was required to remain slightly flexed at the hip and knee, so that the foot was hovering approximately parallel to the mid-shin of the jumping limb, with no swinging allowed	Nil	Practice testing session 1 week before testing	Dynamic stretching and 3 submaximal trials
Troester et al. [57]	Unilateral Drop Landing (from CMJ Height)	In-season	Participants started at a point 1 metre from the centre of the force plate Jump as high as possible off two legs and stick and hold the landing on one leg If the landing foot moved after contact or the opposite foot touched down, trails were discarded	Hard surface	Prior familiarity during regular club monitoring procedures	Nil
Yoshida et al. [68]	CMJ	Nil	Holding a plastic PVC pipe behind the neck	Embedded into the laboratory floor	2 practice testing sessions prior to testing date	Dynamic stretching, linear, and lateral movement drills, such as jogging, skipping, and shuffling
Cabarkapa et al. [69]	CMJ	In-Season	Maximal effort trials, hands on the hips during the entire movement, focus on pushing the ground as explosively as possible	Nil	Demonstration of testing battery on arrival to session	10-15 minutes of dynamic stretching
Cabarkapa et al. [70]	CMJ	In-Season	No arm-swing (i.e., hands on the hips during the entire movement)	Nil	Demonstration of testing battery on arrival to session	15 minute standardised team warm-up routine
Donahue et al. [71]	CMJ	Nil	Self-selected countermovement depth and foot position Jump “as high as possible” A dowel was placed across the upper back	Nil	Nil	10 minutes of dynamic movements and submaximal CMJs
Janicijevic et al. [72]	Bilateral CMJ Unilateral CMJ	N/A	Jump “as high as possible”	Nil	Practice testing session prior to testing date	5 minutes of running at a selected pace
Spencer et al. [73]	CMJ	In-Season	Self-selected countermovement depth Jump “as fast and high as possible” Hands on hips	Solid vinyl-lined	Prior familiarity during regular training	10 minutes of jogging, dynamic mobility, activation of lower-body musculature, and two sub-maximal CMJs
Tazji et al. [74]	Bilateral Hopping	Nil	Maximum jump height and minimum contact time Subjects’ hands were put on their waists and they were barefoot.	Nil	Participants were taught how to hop prior to testing	5 minutes of running and 5 minutes of static stretching
Philipp et al. [75]	CMJ 10/5 RJ	Pre-Season	Hands placed on hips Jump “ as fast and as high as possible”	Nil	Nil	“Static and dynamic warmup”

CMJ, countermovement jump; DJ, drop jump; SJ, squat jump; Nil, information not provided; N/A, not applicable.

Additional considerations for the standardisation of testing were described in some studies, such as a description of the surface used (N = 3; [57,68,73]), details of familiarisation protocol (N = 20; [21,31,32,51,54–58,60,61,64–66,68–70,72–74]), details of prescribed warm-up prior to testing (N = 21; [21,31,32,44,54–56,58,60–62,65,66,68–75]), zeroing of force plates prior to each test (N = 6; [31,44,63,70,73,75]), the process of weighing participants prior to each test trial (N = 6; [21,44,66,71–73]), trials performed (N = 28; [21,31,32,44,50–58,60–64,66–75]), between-trial rest (N = 20; [21,31,32,44,50–56,60,62,68–73,75]), and data used for statistical analysis (N = 24; [21,31,32,44,50–58,60,61,64–66,68,69,71–74]) (Table 3). Many different force plate models were identified (N = 18) across 10 different providers (Table 4). The most frequently used force plate provider was Kistler Instruments Ltd (N = 9; [44,51,53,57,58,60,63,66,72]), followed by Fitness Technology (N = 5; [21,31,32,61,62]), and American Mechanical Technology Inc. (AMTI) (N = 4; [54,56,64,71]) (Table 4). The most commonly used force plate model was the 400 Series Performance Plate (Fitness Technology, Adelaide, Australia) (N = 5; [21,31,32,61,62]) (Table 4). There were 7 different data collection frequencies used, and the most popular was 1000 Hz (N = 17; [44,52,54,55,57,58,60,63–65,68–71,73–75]), the highest was 5000 Hz (N = 1; [50]), and the lowest was 200 Hz (N = 3; [31,32,61]) (Table 4).

**Table 3:** Summary of additional data collection protocols.

Author(s)	Zeroing?	Weighing?	Trials Performed	Between-Trial Rest	Data Used for Analysis
Bedo et al. [50]	Nil	Nil	Baseline: 3 Post: 10	1 minute	Baseline: average of trials Post: individual trials
Boullosa et al. [51]	Nil	Nil	2	15 seconds	Trial with greatest jump height
Lonergan et al. [52]	Nil	Nil	3	1 minute	Average of trials
Lupo et al. [53]	Nil	Nil	2	1 minute	Trial with greatest peak concentric force
Scanlan et al. [54]	Nil	Nil	2	2 minutes	Trial with greatest jump height
McLellan et al. [55]	Nil	Nil	3	3 minutes	Trial with greatest jump height
Thorlund et al. [60]	Nil	Nil	3	30-45 seconds	Trial with greatest jump height
Gathercole et al. [31]	Before every trial	Nil	6	90 seconds	Average of 4 most consistent trials
Kennedy et al. [44]	Before each trial	Average force during the first second of a 2 second quiet standing period	5	1 minute	Average of trials
McLean et al. [61]	Nil	Nil	1	N/A	Single trial
Roe et al. [62]	Nil	Nil	2	1 minute	Nil
Yu et al. [63]	Before each subject	Nil	3	Nil	Nil
Gathercole et al. [32]	Nil	Nil	6	1 minute	Average of 4 most consistent trials
Oliver et al. [64]	Nil	Nil	3	Nil	Trial with greatest flight time
Clarke et al. [65]	Nil	Nil	Nil	Nil	Trial with greatest TOV
West et al. [66]	Nil	An initial 2 second quiet standing phase	1	N/A	Single trial
Merrigan et al. [56]	Nil	Nil	3	30 seconds	Average of trials
Horita et al. [67]	Nil	Nil	3	Nil	Nil
McCall et al. [58]	Nil	Nil	1	N/A	Single trial
Travis et al. [59]	Nil	Nil	Nil	Nil	Nil
Bromley et al. [21]	Nil	An initial 2 second quiet standing phase	3	30 seconds	Average of trials
Troester et al. [57]	Nil	Nil	2	Nil	Average of trials
Yoshida et al. [68]	Nil	Nil	6	1 minute	Average of the 4 most consistent trials
Cabarkapa et al. [69]	Nil	Nil	3	10-15 seconds	Average of trials
Cabarkapa et al. [70]	Before each subject	Nil	3	10-15 seconds	Nil
Donahue et al. [71]	Nil	Initial 1 second of quiet standing	2	30 seconds	Average of trials
Janicijevic et al. [72]	Nil	Average force over first 1.5 seconds of data collection	3	1 min	Average of trials
Spencer et al. [73]	Before each trial	Average force over first 1 second of quiet standing	3	30 seconds	Average of trials
Tazji et al. [74]	Nil	Nil	3 trials of 15 consecutive hops	Nil	Average of trials
Philipp et al. [75]	Before each trial	Nil	3	15-30 seconds	Nil

Nil, information not provided; N/A, not applicable.

**Table 4:** Tally illustrating hardware used.

References	N	Hardware	Sampling Frequency
Gathercole et al. [31]	5	400 Series Performance Plate Fitness Technology Adelaide, Australia	200 Hz
McLean et al. [61]			200 Hz
Gathercole et al. [32]			200 Hz
Roe et al. [62]			600 Hz
Bromley et al. [21]			600 Hz
McCall et al. [58]	3	Model 9260AA6 Kistler Instruments Ltd Winterthur, Switzerland	1000 Hz
Troester et al. [57]			
Janicijevic et al. [72]			Nil
Thorlund et al. [60]	2	Model 9281B Kistler Instruments Ltd Winterthur, Switzerland	1000 Hz
Yu et al. [63]			
Merrigan et al. [56]	2	AccuPower American Mechanical Technology Inc Watertown, MA, USA	1600 Hz
Donahue et al. [71]			1000 Hz
Spencer et al. [73]	2	Hawkin Dynamics Inc. Westbrook, ME, USA	1000 Hz
Philipp et al. [75]			
Cabarkapa et al. [69]	2	ForceDecks VALD Performance Brisbane, Australia	1000 Hz
Cabarkapa et al. [70]			
Horita et al. [67]	2	Nil	Nil
Travis et al. [59]			
Boullosa et al. [51]	1	Quattro Jump Kistler Instruments Ltd Winterthur, Switzerland	500 Hz
Lupo et al. [53]	1	Model *not stated* Kistler Instruments Ltd Winterthur, Switzerland	2048 Hz
Kennedy et al. [44]	1	Model 9286BA Kistler Instruments Ltd Winterthur, Switzerland	1000 Hz
West et al. [66]	1	Model 92866AA Kistler Instruments Ltd Farnborough, United Kingdom	Nil
Scanlan et al. [54]	1	BP400800–2000 American Mechanical Technology, Inc Watertown, MA, USA	1000 Hz
Oliver et al. [64]	1	OR6–5 American Mechanical Technology, Inc Watertown, MA, USA	1000 Hz
Bedo et al. [50]	1	A/D converter #USB-6251-BNC National Instruments Austin, TX, USA	5000 Hz
Lonergan et al. [52]	1	PS 2141 Pasco Roseville, CA, USA	1000 Hz
McLellan et al. [55]	1	ONSPOT 200–1 Innervations Muncie, IN, USA	1000 Hz
Clarke et al. [65]	1	Na4060-10 Bertec Columbus, OH, USA	1000 Hz
Yoshida et al. [68]	1	Rice Lake Weighing Systems Rice Lake, WI, USA	1000 Hz
Tazji et al. [74]	1	Model *not stated* Bertec, United Kingdom	1000 Hz

Hz, hertz; Nil, information not provided.

### Summary of study design

A total of 6 of the 30 studies did not include sufficient results information for their measures (i.e., means and SDs for a metric of each test at each time point), meaning a detailed summary of study design information was only possible for 24 studies [21,31,32,44,51,53–58,60,63–65,67–75]. The assessments utilised in these 24 studies included the CMJ (N = 18; [31,32,44,51,53–55,60,63–65,68–73,75]), DJ (N = 3; [56,64,67]), SJ (N = 1; [64]), unilateral CMJ (N = 2; [21,72]), bilateral hopping (N = 1; [74]), 10/5 RJ test (N = 1; [75]), unilateral drop landing (N = 1; [57]), and the “isometric posterior lower-limb muscle test” (N = 1; [58]) (Table 5).

**Table 5:** Summary of study design.

Author(s)	Test	Activity Performed	Activity Measures	Mean ± SD	Baseline	Post-Intervention
Boullosa et al. [51]	CMJ	Université de Montréal Track Test	Time (s)	1476 ± 145	- 2 min	+ 2 min
			MAS Value (km/h)	18.9 ± 1.2		
Lupo et al. [53]	CMJ	Regular Training Session (Rugby Union)	Total Distance Covered (m)	With Tackles: 5020 ± 253 No Tackles: 6270 ± 216	- 0 min	+ 0 min
			Average Pace (metres/min)	With Tackles: 59.7 ± 1.91 No Tackles: 64.2 ± 1.66		
Scanlan et al. [54]	CMJ	Basketball Exercise Simulation Test	Time (min)	40	- 2 min	+ 2 min
			Rounds Performed	80		
McLellan et al. [55]	CMJ	8 Separate Competitive Matches (Rugby League)	Nil	Nil	- 24 h - 30 min	+ 30 min, + 24 h + 48 h, + 72 h + 96 h, + 120 h
Thorlund et al. [60]	CMJ	Simulated Handball Match	Running (m)	594.3	- 0 min	+ 0 min
			Fast Running (m)	401.25		
			Sprinting (m)	247.00		
			Jump Shots (number)	28		
			Feints (number)	56		
			Total Distance Covered (m)	6527.20		
Gathercole et al. [31]	CMJ	High-intensity Intermittent-exercise Running Test	Total Distance Covered (m)	8613 ± 1249	- 0 min	+ 0 min + 24 h + 72 h
Kennedy et al. [44]	CMJ	3 Regular High-intensity Training Sessions (Rugby Union)	Nil	Nil	- 0 min	+ 24 h + 48 h
Yu et al. [63]	CMJ	Standardised Treadmill Running Test	Nil	Nil	- 0 min	+ 0 min
Gathercole et al. [32]	CMJ	Intermittent High-intensity Stair-climb Fatigue Protocol	Total Time at Maximal Exertion (s)	375	- 30 min	+ 30 min
Oliver et al. [64]	CMJ DJ SJ	Intermittent High-intensity Exercise Test on Non-motorised Treadmill	Total Distance Covered (m)	4745 ± 102	- 0 min	+ 0 min
			Average Heart Rate (bpm)	173 ± 12		
Clarke et al. [65]	CMJ	Canadian Football G-sim	Maximum Heart Rate (bpm)	187.5 ± 9.3	- 0 min	+ 0 min + 24 h + 48 h
Merrigan et al. [56]	DJ	Incremental Treadmill Running Test	Time (min)	7–10	- 2 min	+ 2 min
Horita et al. [67]	DJ	Continuous Rebound Jump Fatigue Protocol on a Sledge Apparatus at 23 Degrees Incline	Repetitions to Exhaustion (number)	117 ± 70	- 0 min	+ 0 min + 2 h + 96 h
McCall et al. [58]	Isometric Posterior Lower-limb Muscle Test	Competitive Match (Soccer)	Nil	Nil	- 15 min	+ 15 min + 1 wk
Bromley et al. [21]	Unilateral CMJ	A Single 90 Minute Competitive Match (Soccer)	Nil	Nil	- 2 h	+ 1 h + 24 h + 72 h
Troester et al. [57]	Unilateral Drop Landing (from CMJ Height)	A Reuglar Training Week and Weekend Competitive Match (Rugby Union)	Nil	Nil	- 0 min	+ 1 wk
Yoshida et al. [68]	CMJ	Fatiguing Exercise Protocol Consisting of Basketball Drills.	Time (min) Session RPE (Borg’s CR-10)	60 7.7 ± 1.0	- 0 min	+ 0 min + 2 h + 6 h + 24 h + 48 h + 72 h
Cabarkapa et al. [69]	CMJ	Regular Court-Based Practice Session	Time (min)	120	- 0 min	+ 0 min
Cabarkapa et al. [70]	CMJ	Regular Court-Based Practice Session	Time (min) Session RPE (Borg’s CR-10)	90 6.60 ± 1.17	- 0 min	+ 0 min
Donahue et al. [71]	CMJ	Sport-Specific Volleyball Training Session	Time (min) Jump Count (average) Jump Count (range)	120 103.66 81–165	- 0 min	+ 0 min
Janicijevic et al. [72]	Bilateral CMJ Unilateral CMJ	Unilateral and Bilateral Knee Extension Fatigue Protocol	Repetitions to Failure	Nil	- 0 min	+ 0 min
Spencer et al. [73]	CMJ	Competitive Fixture (Soccer)	Time Played (min) Total Distance (m) Sprint Distance (m) Average Speed (m/min) Top Speed (m/s) Power plays (count)	83.13 ± 11.32 9550 ± 1866 974.27 ± 438.47 98.90 ± 20.41 8.53 ± 0.34 71.29 ± 21.95	- 90 min	+ 15 min
Tazji et al. [74]	Bilateral Hopping	Core Stability Muscle Fatigue Protocol	Time (min) Exercises x Sets x Time	30 8 x 4 x 20	- 0 min	+ 0 min
Philipp et al. [75]	CMJ 10/5 RJ	5-Day High-Intensity Stressful Training Phase	Accumulated Acceleration Load Acceleration Load Medium Acceleration Load High Acceleration Load Very High Anaerobic Activity Distance	3,106 ± 538 479 ± 121 934 ± 133 858 ± 387 11,581 ± 2,825	- 1 week	+ 6 h + 72 h + 1 week

CMJ, countermovement jump; DJ, drop jump; SJ, squat jump; RJ, rebound jump; SD, standard deviation; s, seconds; km, kilometres; wk, week; h, hour; m, metres; min, minute; bpm, beats per minute; Nil, information not provided; MAS, maximal aerobic speed; RPE, rating of perceived exertion.

Of the 24 studies, 8 different baseline (pre-activity) testing timepoints were reported, which were -1 week (N = 1; [75]), –24 h (N = 1; [55]), -2 h (N = 1; [21]), -90 min (N = 1; [73]), –30 min (N = 2; [32,55]), -15 min (N = 1; [58]), –2 min (N = 3; [51,54,56]), and –0 min (N = 15; [31,44,53,57,60,63–65,67–72,74]) (Table 5). Additionally, 13 different post-activity testing timepoints were reported, which were +0 min (N = 13; [32,53,60,63–65,67–72,74]), + 2 min (N = 3; [51,54,56]), + 15 min (N = 2; [58,73]), + 30 min (N = 2; [31,55]), + 1 h (N = 1; [21]), + 2 h (N = 2; [67,68]), + 6 h (N = 2; [68,75]), + 24 h (N = 6; [21,31,44,55,65,68]), + 48 h (N = 4; [44,55,65,68]), + 72 h (N = 5; [21,31,55,68,75]), + 96 h (N = 2; [55,67]), + 120 h (N = 1; [55]), and + 1 week (N = 3; [57,58,75]) (Table 5).

The effects of 6 different activities on NMF were identified, which were categorised as a usual rugby union training microcycle (i.e., a regular week of usual outdoor and indoor training sessions and competitive match-play) (N = 1; [57]), usual competitive match-play only (N = 3 soccer; [21,58,73], N = 1 rugby league; [55]), a usual outdoor (field-based) rugby union training session only (N = 2; [44,53]), a usual indoor (court-based) basketball training session only (N = 2; [69,70]), a pre-determined training session designed to simulate competitive match demands (N = 2 basketball; [54,68], N = 1 handball; [60], N = 1 Canadian football; [65], N = 1 Volleyball; [71]), a pre-determined high-intensity fatigue protocol (N = 4; [32,67,72,74]), a standardised cardiovascular fitness test (i.e., incremental treadmill running test) (N = 5; [31,51,56,63,64]), and a planned 5-day “high-intensity stressful training phase” (N = 1; [75]) (Table 5).

TL was quantified using time (seconds or minutes) (N = 9; [51,54,56,68–71,73,74]), maximal aerobic speed (MAS; km/h) (N = 1; [51]), external TL values (i.e., total distance covered, running, fast running, and sprinting distance; m) (N = 6; [31,53,60,64,73,75]), average speed (metres/min) (N = 2; [53,73]), number of rounds performed (N = 1; [54]), number of sport-specific actions performed (i.e., jump shots and feints) (N = 2; [60,71]), repetitions performed to volitional exhaustion (N = 2; [67,72]), internal TL values (i.e., average and maximum heart rate [HR]; bpm) (N = 4; [64,65]), and total time at maximal exertion (s) (N = 1; [32]). Of these 16 studies, TL was not quantified in 7 studies ([21,44,55,57,58,63,72]; Table 5).

### Summary of measures

As described above, a detailed summary of measures used was only possible for the 24 studies which presented sufficient results information (Tables 5–9), where 90 different metrics were reported across the 18 studies which utilised the CMJ (Table 6), which equates to an average of 5 metrics per study. The top 5 most frequently reported metrics across these studies, in order of most to least frequent, were JH (N = 14; [31,32,44,51,54,60,63,68–73,75]), concentric phase time (N = 7; [31,32,44,60,68–70]), eccentric phase time (N = 7; [31,32,44,60,68–70]), peak force (N = 7; [31,32,44,51,55,64,65]), and peak power (N = 6; [31,32,44,51,55,65]) (Table 6).

**Table 6:** Tally illustrating countermovement jump metric selection and calculation methods.

References	Test	Metric	N	Calculation
Cabarkapa et al. [69]	CMJ	mRSI (AU)	5	Jump height divided by time to take-off.
Donahue et al. [71]	CMJ			
Spencer et al. [73]	CMJ			
Philipp et al. [75]	CMJ			
Cabarkapa et al. [70]	CMJ			Not described.
Kennedy et al. [44]	CMJ	Flight Time Alternative Contraction Time Ratio (AU)	1	The ratio of flight time to alternative contraction time. Alternative contraction time was determined by calculating the peak relative eccentric power and data points within a 10% range on the power-time trace, and then completing a backward search of consecutive time points until the power change is < 0.15 W/kg for more than 4 out of 5 consecutive pairs.
Gathercole et al. [31]	CMJ	Flight Time Contraction Time Ratio (AU)	3	The ratio of flight time to contraction time. Contraction time is the duration from jump initiation to take-off.
Kennedy et al. [44]	CMJ			
Yoshida et al. [68]	CMJ			Not described.
Boullosa et al. [51]	CMJ	Jump Height (cm)	14	Height of the center of mass at the apex minus at the point of take-off.
Scanlan et al. [54]	CMJ			Via the flight time method (1/2 gravitational acceleration * (flight time/ 2)^2^).
Thorlund et al. [60]	CMJ			Via the take-off velocity method (TOV^2^/ 2 * gravitational acceleration).
Cabarkapa et al. [69]	CMJ			
Spencer et al. [73]	CMJ			
Philipp et al. [75]	CMJ			
Gathercole et al. [31]	CMJ			The maximum jump height achieved, calculated using peak velocity.
Kennedy et al. [44]	CMJ			
Gathercole et al. [32]	CMJ			
Yu et al. [63]	CMJ			Via the flight time method (gravitational acceleration^-2^ * flight time^2^)/ 8.
Janicijevic et al. [72]	CMJ			
Yoshida et al. [68]	CMJ			Not described.
Cabarkapa et al. [70]	CMJ			
Donahue et al. [71]	CMJ			
Spencer et al. [73]	CMJ	Jump Momentum (m/s·kg)	2	Vertical velocity of the center of mass at take-off multiplied by body mass.
Philipp et al. [75]	CMJ			
Thorlund et al. [60]	CMJ	Take-off Velocity (m/s)	2	Not described.
Clarke et al. [65]	CMJ			Integration of the GRF data, combined with body mass, allowed calculation of the vertical velocity profile, which was used to obtain TOV.
Gathercole et al. [32]	CMJ	Flight Time (s)	2	Time spent in the air from jump take-off to landing.
Oliver et al. [64]	CMJ			The time between when force was < 10 N (instant of take-off) and when force returned to < 10 N (instant of touch-down).
Boullosa et al. [51]	CMJ	Vertical COM displacement (cm)	1	Not described.
Cabarkapa et al. [69]	CMJ	Countermovement Depth (cm)	5	Not described.
Cabarkapa et al. [70]	CMJ			
Donahue et al. [71]	CMJ			Integration of the center of mass velocity data with respect to time provided the center of mass displacement.
Spencer et al. [73]	CMJ			Peak negative value of center of mass displacement prior to take-off.
Philipp et al. [75]	CMJ			Lowest center of mass displacement, transition from braking to propulsive phase.
Thorlund et al. [60]	CMJ	Concentric Displacement (cm)	1	Maximal centre of mass displacement during positive velocity.
Thorlund et al. [60]	CMJ	Eccentric Displacement (cm)	1	Maximal centre of mass displacement during negative velocity.
Gathercole et al. [32]	CMJ	Time to Peak Power (s)	1	Time from jump initiation to peak power.
Yoshida et al. [68]	CMJ	Total Time to Peak Power (s)	1	Not described.
Gathercole et al. [32]	CMJ	Time to Peak Force (s)	1	Time from jump initiation to peak force.
Yoshida et al. [68]	CMJ		1	Not described.
Spencer et al. [73]	CMJ	Time to Take-off (s)	3	Time between the onset of movement and take-off.
Donahue et al. [71]	CMJ			
Philipp et al. [75]	CMJ			Duration from start of the countermovement until take-off.
Yoshida et al. [68]	CMJ	Total Duration (s)	1	Not described.
Oliver et al. [64]	CMJ	Contact Time (s)	1	The period between when force change by more than 10 N from resting body weight (initiation of movement), to when force was < 10 N (instant of take-off).
Gathercole et al. [31]	CMJ	Total Movement Time (s)	4	Time required to perform the entire CMJ (i.e., both eccentric and concentric phases).
Kennedy et al. [44]	CMJ			
Gathercole et al. [32]	CMJ			
Thorlund et al. [60]	CMJ	Take-off Phase Time (s)		Eccentric plus concentric phase time.
Cabarkapa et al. [69]	CMJ	Contraction Time (s)	2	Not described.
Cabarkapa et al. [70]	CMJ			
Philipp et al. [75]	CMJ	Propulsive Phase Time (s)	3	Total duration of propulsive phase.
Spencer et al. [73]	CMJ			Not described.
Donahue et al. [71]	CMJ			Time between the end of the braking phase to the point of takeoff.
Thorlund et al. [60]	CMJ	Concentric Phase Time (s)	7	Total time of positive velocity prior to take-off.
Gathercole et al. [31]	CMJ			Time required to perform the concentric CMJ phase.
Kennedy et al. [44]	CMJ			
Gathercole et al. [32]	CMJ			
Yoshida et al. [68]	CMJ			Time period from zero velocity to takeoff.
Cabarkapa et al. [69]	CMJ			Not described.
Cabarkapa et al. [70]	CMJ			
Donahue et al. [71]	CMJ	Braking Duration (s)	1	Time between the the point at which vertical ground reaction force surpassed the calculated body mass during one second of quiet stance prior to the trial initiation until the instant the center of mass velocity reaches zero.
Philipp et al. [75]	CMJ	Braking Phase Time (s)	4	Total duration of braking phase.
Cabarkapa et al. [69]	CMJ			Not described.
Cabarkapa et al. [70]	CMJ			
Spencer et al. [73]	CMJ			
Thorlund et al. [60]	CMJ	Eccentric Acceleration Time (ms)	1	Interval between the start of downward movement (velocity increases negatively) and the instant of maximal negative velocity.
Thorlund et al. [60]	CMJ	Eccentric Deceleration Time (ms)	1	Interval between maximal negative velocity (i.e., instant that force = body mass) and the time when velocity reached zero (i.e., the end of downward movement).
Thorlund et al. [60]	CMJ	Eccentric Phase Time (s)	7	Total time of negative velocity prior to take-off.
Gathercole et al. [31]	CMJ			Time required to perform the eccentric CMJ phase.
Kennedy et al. [44]	CMJ			
Gathercole et al. [32]	CMJ			
Yoshida et al. [68]	CMJ			Time period from the initiation of the jump to zero power.
Cabarkapa et al. [69]	CMJ			Not described.
Cabarkapa et al. [70]	CMJ			
Yoshida et al. [68]	CMJ	Stretching Phase Duration (s)	1	Not described.
Spencer et al. [73]	CMJ	Unweighting Phase Time (s)	1	Not described.
Gathercole et al. [32]	CMJ	Rate of Power Development (W/s)	1	Largest power increase during a 30 ms epoch.
Gathercole et al. [32]	CMJ	Mean Eccentric and Concentric Power Over Time (W/kg/s)	2	The sum of power produced during both eccentric and concentric CMJ phases, divided by the time taken (in ms) to perform the jump.
Kennedy et al. [44]	CMJ			
Gathercole et al. [31]	CMJ	Mean Eccentric and Concentric Power (W/kg/s)	1	The sum of power produced during both eccentric and concentric CMJ phases.
Thorlund et al. [60]	CMJ	Relative Concentric Peak Power (N/kg)	1	Concentric peak power divided by body mass (kg).
Cabarkapa et al. [69]	CMJ	Concentric Peak Power (W)	2	Not described.
Cabarkapa et al. [70]	CMJ			
Gathercole et al. [31]	CMJ	Relative Peak Power (W/kg)	2	Peak power divided by body mass (kg).
Yoshida et al. [68]	CMJ			
Boullosa et al. [51]	CMJ	Peak Power (W/kg)	6	Greatest instantaneous power produced during the propulsion phase.
McLellan et al. [2012]	CMJ			Peak force was multiplied by the peak velocity in the propulsive phase.
Gathercole et al. [31]	CMJ			Greatest power achieved during the jump.
Kennedy et al. [44]	CMJ			
Gathercole et al. [32]	CMJ			
Clarke et al. [65]	CMJ			Peak force multiplied by peak velocity.
Thorlund et al. [60]	CMJ	Relative Concentric Mean Power (W/kg)	1	Concentric mean power divided by body mass (kg).
Cabarkapa et al. [69]	CMJ	Concentric Mean Power (W)	2	Not described.
Cabarkapa et al. [70]	CMJ			
Gathercole et al. [31]	CMJ	Relative Mean Power (W/kg)	2	Mean power divided by body mass (kg).
Yoshida et al. [68]	CMJ			
Boullosa et al. [51]	CMJ	Mean Power (W/kg)	4	Average power produced during the propulsion phase.
Gathercole et al. [31]	CMJ			Mean power generated during the concentric phase of the jump.
Kennedy et al. [44]	CMJ			
Gathercole et al. [32]	CMJ			
Cabarkapa et al. [69]	CMJ	Eccentric Peak Power (W)	2	Not described.
Cabarkapa et al. [70]	CMJ			
Cabarkapa et al. [69]	CMJ	Eccentric Mean Power (W)	2	Not described.
Cabarkapa et al. [70]	CMJ			
Thorlund et al. [60]	CMJ	Relative Concentric Work (J/kg)	1	Not described.
Thorlund et al. [60]	CMJ	Velocity at Concentric Peak Power (m/s)	1	Not described.
Gathercole et al. [32]	CMJ	Velocity at Peak Power (m/s)	1	The velocity recorded at the time point where peak power occurs.
Cabarkapa et al. [69]	CMJ	Concentric Peak Velocity (m/s)	2	Not described.
Cabarkapa et al. [70]	CMJ			
Gathercole et al. [31]	CMJ	Peak Velocity (m/s)	3	Greatest velocity achieved during the jump.
Kennedy et al. [44]	CMJ			
Gathercole et al. [32]	CMJ			
Gathercole et al. [32]	CMJ	Minimum Velocity (m/s)	1	Lowest jump velocity during the eccentric phase.
Thorlund et al. [60]	CMJ	Concentric Peak Velocity (m/s)	1	Not described.
Philipp et al. [75]	CMJ	Average Braking Velocity (m/s)	1	Average center of mass velocity during the braking phase.
Cabarkapa et al. [69]	CMJ	Eccentric Peak Velocity (m/s)	2	Not described.
Cabarkapa et al. [70]	CMJ			
Gathercole et al. [31]	CMJ	Area Under the Force-Velocity Trace (Ns)	3	The area under the force–velocity trace where eccentric movement is performed (i.e., the area under the left side of the trace).
Kennedy et al. [44]	CMJ			
Gathercole et al. [32]	CMJ			
Gathercole et al. [32]	CMJ	Relative Net Impulse (Ns/kg)	2	Total impulse divided by participant’s body mass (kg).
Yoshida et al. [68]	CMJ			Time period during net impulse divided by body mass (kg).
Donahue et al. [71]	CMJ	Propulsive Net Impulse (Ns)	1	Not described.
Janicijevic et al. [72]	CMJ	Propulsive Impulse (Ns)	1	Product of mean force and the duration of the propulsive phase.
Cabarkapa et al. [69]	CMJ	Concentric Impulse (Ns)	2	Not described.
Cabarkapa et al. [70]	CMJ			
Yoshida et al. [68]	CMJ	Positive Impulse (Ns)	1	Time period during the vGRF exceeds system weight.
Gathercole et al. [32]	CMJ	Total Impulse (Ns)	1	Force exerted concentrically multiplied by the time taken concentrically.
Philipp et al. [75]	CMJ	Braking Net Impulse (Ns)	1	Net vertical impulse during the braking phase.
Donahue et al. [71]	CMJ	Braking Impulse (Ns)	3	Not described.
Cabarkapa et al. [69]	CMJ			
Cabarkapa et al. [70]	CMJ			
Yoshida et al. [68]	CMJ	Unweighted Impulse (Ns)	1	Not described.
Boullosa et al. [51]	CMJ	Stiffness (N/m/kg)	1	Not described.
Thorlund et al. [60]	CMJ	Relative RFD 0–100 ms (N/s/kg)	1	Maximal vertical force achieved within 100 ms divided by 100 ms, divided by body mass.
Thorlund et al. [60]	CMJ	Relative RFD 0–50 ms (N/s/kg)	1	Maximal vertical force achieved within 50 ms divided by 50 ms, divided by body mass.
Yoshida et al. [68]	CMJ	Average Rate of Force Development (N/s)	1	Not described.
Philipp et al. [75]	CMJ	Braking RFD (N/s)	1	Average change in force over time during the braking phase.
Yoshida et al. [68]	CMJ	Stretching Phase Rate of Force Development (N/s)	1	Not described.
McLellan et al. [55]	CMJ	RFD (N/s)	2	The maximum force that occurred over the “first derivative” of the force-time curve.
Gathercole et al. [32]	CMJ			Largest force increase during a 30 ms epoch.
Thorlund et al. [60]	CMJ	Relative Force at Concentric Peak Power (N/kg)	1	Not described.
Yu et al. [63]	CMJ	Landing Peak Vertical Force (N)	1	Not described.
Gathercole et al. [31]	CMJ	Force at 0 Velocity (N)	3	The force exerted at the end of the countermovement where the jump transitions from eccentric to concentric movement (i.e., velocity is at zero).
Kennedy et al. [44]	CMJ			
Gathercole et al. [32]	CMJ			
Yu et al. [63]	CMJ	Push-off Peak Vertical Force (N)	1	Not described.
Lupo et al. [53]	CMJ	Concentric Peak Force (N)	4	Maximal vertical force during the concentric phase (between the instant that the center-of-mass velocity exceeded 0.01 m/s − 1 and the instant of takeoff (i.e., when the vertical ground reaction force fell below 5 times the SD of the flight phase force).
Thorlund et al. [60]	CMJ			Maximal vertical force during the concentric phase (when velocity was positive).
Cabarkapa et al. [69]	CMJ			Not described.
Cabarkapa et al. [70]	CMJ			
Gathercole et al. [31]	CMJ	Relative Peak Force (N/kg)	2	Peak force divided by body mass (kg).
Yoshida et al. [68]	CMJ			
Boullosa et al. [51]	CMJ	Peak Force (N)	7	Greatest vertical force produced during the propulsion phase.
McLellan et al. [55]	CMJ			Maximum vertical ground reaction force achieved.
Gathercole et al. [31]	CMJ			Greatest force achieved during the jump.
Kennedy et al. [44]	CMJ			
Gathercole et al. [32]	CMJ			
Oliver et al. [64]	CMJ			Greatest force generated during the propulsion phase.
Clarke et al. [65]	CMJ			Maximum vertical ground reaction force achieved.
Thorlund et al. [60]	CMJ	Relative Concentric Mean Force (N/kg)	1	Concentric peak force divided by body mass (kg).
Cabarkapa et al. [69]	CMJ	Concentric Mean Force (N)	2	Not described.
Cabarkapa et al. [70]	CMJ			
Gathercole et al. [31]	CMJ	Relative Mean Force (N/kg)	2	Mean force divided by body mass (kg).
Yoshida et al. [68]	CMJ			
Gathercole et al. [31]	CMJ	Mean Force (N)	5	Mean force generated during the concentric phase of the jump.
Kennedy et al. [44]	CMJ			
Gathercole et al. [32]	CMJ			
Oliver et al. [64]	CMJ			Mean force generated during the propulsion phase.
Janicijevic et al. [72]	CMJ			
Donahue et al. [71]	CMJ	Propulsive Mean Force (N)		Propulsive mean force minus body mass.
Oliver et al. [64]	CMJ	Propulsive Force (N)	1	Not described.
Cabarkapa et al. [69]	CMJ	Eccentric Peak Force (N)	2	Not described.
Cabarkapa et al. [70]	CMJ			
Thorlund et al. [60]	CMJ	Relative Eccentric Peak Force (N/kg)	1	Eccentric peak force divided by body mass (kg).
Donahue et al. [71]	CMJ	Braking Mean Force (N)	1	Braking mean force minus body mass.
Cabarkapa et al. [69]	CMJ	Eccentric Mean Force (N)	2	Not described.
Cabarkapa et al. [70]	CMJ			
Oliver et al. [64]	CMJ	Braking Force (N)	1	Not described.
Spencer et al. [73]	CMJ	Body Mass (kg)	2	Mean force over the first 1 s of the recorded trials while the participants is standing still and upright divided by gravitational acceleration (9.81 m/s^2^).
Philipp et al. [75]	CMJ			System mass gathered during weighing phase prior to jump.

CMJ, countermovement jump; SD, standard deviation; mRSI, modified reactive strength index; TOV, take-off velocity; RFD, rate of force development; cm, centimetres; m, metres; s, seconds; N, Newtons; kg, kilograms; J, Joules; W, Watts; AU, Arbitrary Unit.

Varying calculations were identified for metrics such as JH (N = 5), peak force (N = 5), peak power (N = 4), eccentric phase time (N = 3), concentric phase time (N = 3), peak concentric force (N = 2), rate of force development (RFD; N = 2), and FT (N = 2) across studies (Table 6). The same metric calculation was utilised for different metric definitions across studies, such as the greatest vertical force produced during the propulsion phase of the CMJ test was defined as peak force (N = 2; [51,64]) and peak concentric force (N = 2; [53,60]) in separate studies (Table 6). The time required to perform both the eccentric and concentric phases of the CMJ tests was defined as total movement time [31,32,44] and take-off phase time [60] in separate studies (Table 6).

For the DJ, 15 different metrics were reported across the 3 studies which utilised the test (average of 5 metrics per study) (Table 7). The measures which were reported more than once across these studies were peak force (N = 2; [56,64]) and contact time (N = 2; [56,64]) (Table 7). A metric calculation was not provided for 9 DJ metrics (Table 7). Varying calculations were identified only for contact time (N = 2; [56,64]) (Table 7). For the 10/5 RJ test, the single study which utilised the test reported 11 metrics ([75]; Table 7). The single study which utilised the bilateral hopping test only utilised one metric, which was stiffness ([74]; Table 7).

**Table 7:** Tally illustrating rebound jump metric selection and calculation methods.

References	Test	Metric	N	Calculation
Merrigan et al. [56]	DJ	RSI (AU)	1	Flight time divided by contact time.
Philipp et al. [75]	10/5 RJ	mRSI (AU)	1	Jump height divided by time-to-takeoff.
Merrigan et al. [56]	DJ	Jump Height (cm)	2	Via the flight time method (1/2 gravitational acceleration * (flight time/ 2)^2^).
Philipp et al. [75]	10/5 RJ			Maximal jump height via impulse—momentum calculation.
Oliver et al. [64]	DJ	Flight Time (ms)	1	The time between when force was < 10 N (instant of take-off) and when force returned to < 10 N (instant of touch-down).
Philipp et al. [75]	10/5 RJ	Jump Momentum (m/s·kg)	1	Vertical center of mass take-off velocity multiplied with athlete body weight.
Horita et al. [67]	DJ	Take-off Velocity (m/s)	1	Not described.
Horita et al. [67]	DJ	Knee Positive Peak Power (W/kg)	1	Not described.
Philipp et al. [75]	10/5 RJ	Average Braking Velocity (m/s)	1	Average center of mass velocity during the braking phase.
Merrigan et al. [56]	DJ	Impulse (Ns)	1	Area under the force-time curve.
Philipp et al. [75]	10/5 RJ	Braking Net Impulse (Ns)	1	Net vertical impulse during the braking phase.
Horita et al. [67]	DJ	Concentric Stiffness (N/m/kg)	1	Not described.
Horita et al. [67]	DJ	Initial Stiffness (N/m/kg)	1	Not described.
Tazji et al. [74]	Bilateral Hopping	Stiffness (N/m/kg)	1	Dividing the maximum vertical ground reaction force by the downward displacement of the center of mass during the ground-contact phase.
Merrigan et al. [56]	DJ	RFD (N/s)	1	Change in vertical ground reaction force from contact to 20 ms after contact divided by 20 ms.
Philipp et al. [75]	10/5 RJ	Braking RFD (N/s)	1	Average change in force over time during the braking phase.
Oliver et al. [64]	DJ	Impact Force (N)	1	Not described.
Merrigan et al. [56]	DJ	Peak Force (N)	2	Maximal vertical ground reaction force.
Oliver et al. [64]				Not described.
Oliver et al. [64]	DJ	Mean Force (N/kg)	1	Not described.
Oliver et al. [64]	DJ	Propulsive Force (N)	1	Not described.
Oliver et al. [64]	DJ	Braking Force (N)	1	Not described.
Philipp et al. [75]	10/5 RJ	Countermovement Depth (cm)	1	Lowest center of mass displacement, transition from braking to propulsive phase.
Merrigan et al. [56]	DJ	Contact Time (ms)	2	Duration from contact (when forces were > 5 SDs above the one-second quite weighing phase average) to takeoff (when forces were < 5 SDs of the quite weighing phase).
Oliver et al. [64]				The period between when force change by more than 10 N from resting body weight (initiation of movement), to when force was < 10 N (instant of take-off).
Philipp et al. [75]	10/5 RJ	Time to Take-off (s)	1	Duration from start of the countermovement until take-off.
Philipp et al. [75]	10/5 RJ	Propulsive Phase Duration (s)	1	Total duration of propulsive phase.
Philipp et al. [75]	10/5 RJ	Braking Phase Duration (s)	1	Total duration of braking phase.
Philipp et al. [75]	10/5 RJ	Body Mass (kg)	1	System mass gathered during weighing phase prior to jump.

DJ, drop jump; RJ, rebound jump; SD, standard deviation; RSI, reactive strength index; mRSI, modified reactive strength index; rate of force development; cm, centimetres; m, metres; s, seconds; AU, arbitrary unit; N, Newtons; kg, kilograms; W, Watts; ms, milliseconds.

For the SJ, the single study which utilised the test reported 5 metrics ([64]; Table 8). A metric calculation was not provided for 3 SJ metrics (Table 8). For the unilateral assessments, 13 different metrics were reported across the 4 studies which utilised the test (average of 3.25 metrics per study), and all measures only featured once across these studies (Table 9). The single study which utilised the “isometric posterior lower-limb muscle test” only utilised one metric, which was peak force ([58]; Table 9).

**Table 8:** Tally to illustrate squat jump metric selection.

References	Test	Metric	N	Calculation
Oliver et al. [64]	SJ	Flight Time (ms)	1	The time between when force was < 10 N (instant of take-off) and when force returned to < 10 N (instant of touch-down).
Oliver et al. [64]	SJ	Peak Force (N)	1	Not described.
Oliver et al. [64]	SJ	Mean Force (N)	1	Not described.
Oliver et al. [64]	SJ	Propulsive Force (N)	1	Not described.
Oliver et al. [64]	SJ	Contact Time (ms)	1	The period between when force change by more than 10 N from resting body weight (initiation of movement), to when force was < 10 N (instant of take-off).

SJ, squat jump; N, Newtons; ms, milliseconds.

**Table 9:** Tally illustrating unilateral assessments metric selection and calculation methods.

References	Test	Metric	N	Calculation
Bromley et al. [21]	Unilateral CMJ	Jump Height (m)	2	Jump height was calculated using the velocity at take-off.
				Dominant Leg Determination Not Described.
Janicijevic et al. [72]				Via the flight time method (gravitational acceleration^-2^ * flight time^2^)/ 8.
				Dominant Leg Determination Not Described.
Bromley et al. [21]	Unilateral CMJ	Landing Impulse (Ns)	1	The sum of impulse on landing up until peak landing force.
				Dominant Leg Determination Not Described.
Bromley et al. [21]	Unilateral CMJ	Concentric Impulse (Ns)	1	The sum of impulse from the end of the braking phase up until take-off.
				Dominant Leg Determination Not Described.
Bromley et al. [21]	Unilateral CMJ	Eccentric Impulse (Ns)	1	The sum of impulse from the end of unweighting period up until the end of the braking phase.
				Dominant Leg Determination Not Described.
Janicijevic et al. [72]	Unilateral CMJ	Propulsive Impulse (Ns)	1	Product of mean force and the duration of the propulsive phase.
				Dominant Leg Determination Not Described.
Janicijevic et al. [72]	Unilateral CMJ	Mean Force (N)	1	Average value of force recorded during the propulsive phase.
				Dominant Leg Determination Not Described.
Bromley et al. [21]	Unilateral CMJ	Peak Landing Force (N)	1	Maximum force obtained during the landing phase of the jump.
				Dominant Leg Determination Not Described.
Bromley et al. [21]	Unilateral CMJ	Peak Propulsive Force (N)	1	Maximum force obtained during the propulsive phase of the jump.
				Dominant Leg Determination Not Described.
McCall et al. [58]	Isometric posterior lower-limb muscle test	Peak Force (N)	1	The maximum ground reaction force achieved.
				Dominant Leg Determination Not Described.
Troester et al. [57]	Unilateral Drop Landing	Time To Stabilisation (s)	1	The time required for force to equalise within 5% of baseline.
				Dominant Leg Determined as Kicking Leg.
Troester et al. [57]	Unilateral Drop Landing	Sway Velocity (cm·s)	1	Total displacement of the centre of pressure divided by the duration of the trial.
				Dominant Leg Determined as Kicking Leg.
Troester et al. [57]	Unilateral Drop Landing	Impulse (Ns)	1	Not described.
				Dominant Leg Determined as Kicking Leg.
Troester et al. [57]	Unilateral Drop Landing	Peak Force (N)	1	Not described.
				Dominant Leg Determined as Kicking Leg.

CMJ, countermovement jump; N, Newtons; m, metres; s, seconds; kg, kilograms; cm, centimetres.

Varying terminology for phase definitions [76] were identified within studies, such as braking (N = 15), eccentric (N = 13), propulsive (N = 10), and concentric (N = 19) used across all tests (Tables 5–8).

## Disussion

The purpose of this scoping review was to identify and describe previous practices of monitoring acute changes in NMF using force plates. Following the application of the search criteria, 30 studies involving the acute monitoring of NMF using force plates were identified [21,31,32,44,50–75]. These 30 studies were qualitatively assessed to determine the characteristics of the studies methodologies. There was a prominent lack of consistency in study methodology, including subject characteristics, force plate hardware used, tests prescribed, metrics calculated from the force-time data (including data analysis procedures), metric terminology used, activity performed, and testing timepoints across studies monitoring acute changes in NMF using force plates. Only 24 of the 30 studies included sufficient results information of a point measure and measure of variability (e.g., mean and SD) for each measure at each timepoint [21,31,32,44,51,53–58,60,63–65,67–75]. Thus, only these 24 studies could be used to collate metric and study design information.

### Subject demographics

The average sample for the studies included in this review was 14.5 ± 5.7 participants per study, with an average age, height, and body mass of 23.2 ± 4.4 years, 181.7 ± 7.6 cm, and 82.4 ± 15.6 kg, respectively. Although a discussion of the required sample size for within-group repeated measures design studies is beyond the scope of this review, the determination of required sample size prior to the commencement of such studies is recommended, to guide researchers towards an acceptable statistical power of results. Of the included studies, male participants featured in 19 studies, while female participants only featured in 3 studies. Of the total 319 participants, males made up 80.25% (N = 256) of participants, while females made up 9.71% (N = 31) of participants. A similar discrepancy was seen in a recent scoping review by Kryger et al. [77] which aimed to characterise the research available on women’s soccer within the health sciences (i.e., sports medicine, strength and conditioning, and sociology) literature. The review identified a distinct gap in the volume of research between males and females, with an initial PubMed search (conducted on 12th June 2020) resulting in a total output of 587,269 results with the key words soccer OR football AND male OR men* OR boy*, as opposed to an output of 4,393 studies for the key words football OR soccer AND female* OR woman OR women OR ladies OR lady [77]. Although it is important to note that the quality and relevance of research articles is more important than the quantity of the return of a search, this preliminary search performed by the authors highlights a clear gap between sexes in the quantity of soccer-related research available [77].

A recent scoping review on the methods used to evaluate physical preparedness longitudinally in the football codes (i.e., soccer, rugby, American football, and Australian rules football, etc.) concluded that only 1 out of 31 articles involved female participants [12]. This discrepancy is concerning as previous researchers have identified that specific outcome and strategy metrics of force plate tests (e.g., the CMJ) distinguish between sexes [78]. In a study by McMahon et al. [78] assessing sex differences in the CMJ, men performed a higher JH through applying greater propulsive impulse and TOV. This was achieved with men performing the task with a greater COM displacement but with a similar movement time to females [78]. Based on this example, it would be negligent to compare female CMJ performance to the more common male benchmarks provided in literature. This consideration also extends to other physical parameters, such as training history, body mass, and strength [79]. With the rise in development of innovative force plate monitoring strategies, the increase in their implementation in men’s and women’s sports [80,81], and the recent increased participation and global coverage of women’s sports [79], diversifying the literature to be more inclusive of all sexes is warranted to encourage and enhance evidence-based practice across all athlete populations. Additionally, without this, a comparison of practical results to published peer-reviewed research is not possible (e.g., normative data in benchmarking) for certain populations. Finally, 3 of the studies included in the current scoping review did not specify their participants’ sex, making up for a total of 10.03% (N = 32) of participants, which is greater than the identified total of female participants, and is unfavourable in terms of research quality.

### Data collection protocols

The standardisation and consistent implementation of force plate data collection protocols is critical to the fitness testing process to allow for accurate and reliable comparisons of data [82]. For example, the preferable surface used for force plate testing would be flat and solid (e.g., concrete) to prevent any unwanted deviations in raw GRF, that might occur due to unlevel force plates or cushioned flooring [82]. Despite this, only Troester et al. [57], Yoshida et al. [68], and Spencer et al. [73] reported details regarding the surface used out of the 30 studies included in this review (Table 2). Once the force plate testing system placement is standardised, data collection protocols must also be appropriate and consistent across sessions. Familiarisation and warm-up protocols were reported in the majority of studies included in this review (N = 20, and N = 21, respectively) (Table 2). It is important that details of familiarisation and warm-up protocols are reported in literature to provide confidence that the presented data represents “maximal” NMF during trials. This is especially important in test-retest reliability studies, where familiarisation is advised prior to any force plate testing, because a lack of it in a specific task can result in inconsistencies between sessions [83]. Thus, it is recommended that details of familiarisation and warm-up protocols are provided in future studies. The zeroing of force plates between trials is also important, as a failure to do so over many trials can cause integration drift leading to erroneous data. Additionally, appropriate processes for weighing athletes during trials is critical, as fluctuations in body weight due to inconsistencies in weighing during VJ trials would compromise the reliability of metrics calculated via forward dynamics, specifically related to acceleration, velocity, and displacement [12]. For the most accurate and reliable data, these factors must be appropriately standardised and repeated between sessions, therefore, it is concerning that of the 30 studies included in this review, the processes of weighing athletes (N = 6) and the zeroing of force plates (N = 6) was scarcely reported (Table 3).

The standardisation of verbal instructions and trial technique is also vital to achieving accurate and reliable force-time data [82]. Jidovtseff et al. [43] reported that the verbal cues given to subjects affects the force-time characteristics, countermovement depth adopted, movement time performed, and the resultant outcome of VJ tasks. Most studies included in this review (N = 24) provided information regarding the utilised verbal cues, with the most common including “jump as high as possible” (N = 12) and perform trials to a “self-selected countermovement depth” (N = 14) (Table 2). Such a technique has been described to promote a more “compliant” strategy, characterised by applying impulse throughout a longer movement time to achieve greater TOV and JH [43]. It makes sense that technique to encourage greater JH has been applied most frequently given that JH was the most frequently reported measure for monitoring acute changes in NMF. However, a combination of ratio, outcome, strategy, and kinetic metrics might provide a better view of overall NMF [84], therefore, other verbal cues (e.g., “jump as fast and high as possible”) could be more suitable. The standardisation of AS within- and between-sessions is also important because utilising an AS has been shown to augment JH [85], but has demonstrated slightly worse measurement reliability than VJs performed without, potentially owing to the increased potential for variation in technique [86]. All studies included in this review which provided details of AS standardisation reported an instruction to perform VJ trials with “hands-on-hips” (N = 15) (Table 2). Regardless of the approach prescribed, consistency in VJs performed with or without AS is required to avoid unwanted alterations in the force-time characteristic and outcome of VJ trials between subjects and sessions.

### Hardware

To the author’s knowledge, the results of this review are the first to illustrate the range of force plate manufacturers and models utilised in research for assessing acute changes in NMF (Table 4). Silva et al. [87] performed a scoping review aiming to map the methodologies used in research for analysing human movement among healthy adolescents. The review reported the use of kinetic data only once out of the 10 studies which met the inclusion criterion, which was conducted by Pau et al. [88] using force plates. Silva et al. [87] concluded that the lack of collection of kinetic data across these studies was likely to do with the feasibility of testing in a “real-world” environment [87]. In this review, a total of 18 different force plate models were reported across the 30 studies included in this review. The most common force plate manufacturer was Kistler Instruments Ltd (N = 9), with 6 different models reported, who are known for producing force plate models which utilise piezoelectric load cells, such as the model “92866aa” utilised in the study by West et al. [66] (Table 4). AMTI were also reported multiple times in this review (N = 3), who typically produce force plate models which utilise strain gauge load cells, such as the Model Biomechanics Measurement Series 400800 utilised by Scanlan et al. [54]. Strain-gauge load cells are also used in the 400 Series Performance Plate by Fitness Technology, which was the most frequently reported force plate model in this review (N = 5) [21,31,32,61,62]. Despite this, two different sampling frequencies were used for the 400 Series Performance Plate across studies, which included 200 Hz [31,32,61] and 600 Hz [21,62] to collect VJ force-time data. These are both lower than the minimum sample frequency of 1000 Hz which has been recommended for the collection of force plate data in VJ tasks [89,90].

Force plate models can be limited in their sampling frequency capability, therefore, an appropriate model must be chosen and consistently applied across testing sessions [82]. In this study, sampling frequency was one of the more well reported data collection protocols (N = 27 out of 30), however, a range of different data collection frequencies was utilised across studies (N = 7) (Table 4). Understandably, the most popular data collection frequency utilised was 1000 Hz (N = 17), with 5000 Hz (N = 1) and 200 Hz (N = 3) being the highest and lowest frequencies reported, respectively (Table 4). The selection of a force plate system to test specific fitness qualities also depends upon accessibility, feasibility, and affordability [82]. A recent study comparing a wireless (Hawkin Dynamics [HD] Inc. 3rd Generation, model 0484) vs in-ground, wired, laboratory based, “gold standard” (AMTI Model Biomechanics Measurement Series 400600) strain-gauge force plate system concluded that the wireless and portable HD Inc. system is as accurate as the less feasible, in-ground, “gold standard”, AMTI force plate system, and thus can be considered as a more feasible option for real-world practice [1]. Additionally, the emergence of integrated proprietary software makes such systems much more feasible for real-world practice as they provide coaches with the ability to immediately and appropriately analyse, interpret, and act upon testing data which could explain the more recent growing body of practice based force plate research [2]. This is positive for future practice and research looking to utilise an accurate yet feasible force plate system for monitoring acute changes in NMF in professional sports settings [1]. With the HD Inc. system being a relatively newer wireless system which has been validated against a traditional “gold standard” system in published research [1], it featured twice in the more recent studies included in this review, specifically, by Spencer et al. [73] and Philipp et al. [75].

### Study design

To the author’s knowledge, the results of this review are the first to illustrate the range of study designs in research concerning monitoring acute changes in NMF using force plates (Table 4). Most research on monitoring acute changes in NMF in sports populations (N = 26) occurred during the in-season period (N = 17) compared to the pre-season period (N = 3) in their respective sports, but several studies did not provide this information (N = 6) which is unfavourable in terms of research (Table 2). A portion (N = 10) of the monitoring in sports populations was conducted around a typical sports schedule, such as a competitive match only (N = 4), a regular training session only (N = 5), and across a regular training week (consisting of multiple training sessions and a competitive match) (N = 1) (Table 5). These findings are understandable given that the rationale for monitoring acute changes in NMF during the competitive (i.e., in-season) period would be to aid in the planning of recovery and the optimisation of physical preparedness for competition, particularly in team sports [79] with congested fixture schedules (e.g., soccer) [91–93]. Besides this, much of the monitoring was employed around standardised activity regimes, such as simulated match-play (N = 3), pre-determined fatigue protocols (N = 5), or a laboratory-based, treadmill, incremental cardiovascular fitness tests (N = 4) (Table 5). Although absolute physical output can be better controlled in these settings, the use of laboratory-based data collection procedures may not transfer to real-world settings [79], thus, it is encouraged that future research that aims to inform practice in competitive sports is performed in real-world competitive sports scenarios and environments.

Quantifying measures of TL is useful as it provides objective information regarding athlete locomotion and an individual’s physiological response to it [94]. External TL values were the most frequently utilised in studies monitoring acute changes in NMF (N = 11). Similarly, Guthrie et al. [12] reported an abundance of studies (N = 11) evaluating longitudinal changes in physical preparedness which utilised external workload measures. The most common measures presented in the review by Guthrie et al. [12] were total distance covered (N = 2), “high-speed” (4.2 to 5.8 m/s) distance covered (N = 13), “very high-speed” (5.5 to 6.4 m/s) distance covered (N = 2), and “sprint” (>6.7 m/s) distance covered (N = 1), whilst one article quantified external workload as total competitive match minutes [12]. This review identified that total distance covered (m) was the most frequently utilised in research monitoring acute changes in NMF (N = 5). External workload data is often accompanied by internal workload (sometimes referred to as “intensity”) data, which is commonly measured through players wearing devices which monitor changes in HR [53]. A combination of external and internal workload measures should be used as an indicator of overall TL, because an individual’s physiological response to the external workload performed during training and matches can differ between athletes [53]. The findings of this study mirror exactly the findings of Guthrie et al. [12], where internal workload measures were utilised less frequently (N = 2) than external workloads, and solely assessed via HR measures, with average [64] and maximum [65] HR utilised once in separate studies.

Potentially the most important factor in monitoring acute changes in NMF is determining the testing time-points to be employed. Fatigue mechanisms work in different combinations, magnitudes, and timeframes throughout competition and during the recovery process [95]. For example, towards the end of and immediately after a competitive soccer match, neuromuscular fatigue is present due to the confounding impacts of mechanical processes (e.g., muscle damage), bioenergetic processes (e.g., lowered muscle glycogen), metabolic processes (e.g., lactate accumulation and acidity), and their effect on neural processes (e.g., disturbances in muscle ion homeostasis and an impaired excitation of the sarcolemma) [95–97]. Of the various baseline testing timepoints reported in this review (N = 8), immediately (<15 min) prior to activity was the most frequent (N = 19) (Table 5). A greater variation of testing timepoints post-activity was seen (N = 13), but immediately (<15 min) post-activity was also prescribed most frequently (N = 18) (Table 5). These findings are understandable given that testers would wish to mitigate any confounding factors in-between testing and the activity of interest which could affect the true determination of neuromuscular fatigue in response to a specific activity. Once an athlete returns to training (usually within 72 hours post-match) [6], the accumulation of mechanical, neural, bioenergetic, and metabolic fatigue would likely create a delayed training effect, delayed onset of muscle soreness, and resultant impairment of physical capacity [6]. This might explain why, second to immediately post-activity testing, the most common testing timepoints reported were +24 h (N = 6), + 48 hours (N = 4), and + 72 h (N = 5), which as stated, might represent when athletes return to training and the days following that in these studies, where the monitoring of recovery and adaptation are warranted (e.g., before players commence preparations for the next match). Understanding that an impairment in NMF at different testing timepoints is due to differing physiological factors can also help practitioners to prescribe appropriate recovery strategies.

### Tests

In various sports, dynamic force plate tests are employed as an objective measure of a specific task performed in competition (e.g., jumping to perform a block in basketball), or as an indicator of lower-body NMF which relates to other sports specific tasks (e.g., in sprinting and changing direction) [82]. When determining a suitable dynamic test, it should be considered if tests are to be performed unilaterally or bilaterally, with or without a countermovement, and vertically or horizontally [82]. Guthrie et al. [12] illustrated that dynamic tests (N = 14) were more popular than isometric tests (N = 2) in research evaluating longitudinal changes in physical preparedness. Of these dynamic tests, all were vertically orientated (i.e., VJ tests), where the bilateral CMJ was the most frequently utilised (N = 11), followed by the bilateral SJ (N = 2), and the unilateral CMJ test (N = 1) [12]. The results of the present review demonstrate many similarities to those of Guthrie et al. [12], with a proportionally greater use of dynamic assessments (N = 33) compared to isometric assessments (N = 2) in research monitoring acute changes in NMF (Table 2). Of these dynamic assessments, all were also vertically orientated (i.e., VJ tests), and the majority (N = 29) were bilateral in nature, where unilateral dynamic assessments featured to a lesser extent (N = 4) (Table 2). The same as the findings of Guthrie et al. [12], the most popular VJ assessments identified in this review were performed with a countermovement, including the slow SSC (i.e., “ballistic”) CMJ test (N = 23), followed by the fast SSC (i.e., “plyometric”) DJ test (N = 3), whilst the concentric-only SJ test was reported only once (Table 2).

Guthrie et al. [12] considered that the popularity of utilisation of the bilateral and vertically orientated CMJ test as an indicator of ballistic lower-body NMF might be due to its ease of application, as it requires minimal familiarity, skill, equipment, and time to complete the test, as well as having a low mechanical and metabolic demand (and thus fatigue and risk of injury). This makes the CMJ test well suited to testing both beginner and more advanced individuals and groups of athletes with ease [82]. Along with this feasibility, a plethora of temporal phase kinetic and kinematic metrics can be calculated from a CMJ force-time curve to provide a detailed overview of lower-body NMF [76]. Isometric tests offer an alternative option to dynamic tests which also demonstrate high feasibility and low mechanical and metabolic demand, which also makes them well suited to testing both beginner and more advanced athletes [82]. However, conducting isometric tests often requires specific equipment configurations (e.g., a custom made IMTP or isometric squat rig) [59], and many tests are performed unilaterally [58], thus often making data collection more complicated, lengthy, and less favourable for practitioners working with large groups of athletes. This could explain why the frequency of utilisation of isometric assessments in research monitoring acute changes in NMF was minimal (N = 2) (Table 2), where tests such as the “isometric posterior lower-limb muscle test” [58] and “isometric squat” [59] featured only once in separate studies (Table 2). These findings mirror those of Guthrie et al. [12], who also reported only two studies which utilised isometric assessments (both utilising the IMTP test), compared to 14 studies which utilised dynamic tests to evaluate long-term changes in physical preparedness following training programmes [12].

### Metrics

The results of this review have identified an abundance of metrics across various tests that have been calculated from force-time data to inform practitioners of acute changes in NMF (Tables 5–9). Out of the metrics reported in these studies, the greatest variety was seen in CMJ metrics (N = 90), where combinations of ratio, outcome, strategy, and kinetic metrics were used to monitor acute changes in NMF (Table 6). Despite CMJ outcome measures showing minimal variety (N = 4), JH was the most frequently reported of all CMJ metrics (N = 14) (Table 6), which corresponds with the findings of Guthrie et al. [12] who reported JH was the most frequently utilised metric used as an indicator of changes in physical preparedness longitudinally (N = 9) [12]. A benefit to utilising force plate systems to monitor NMF is they offer the potential to calculate a vast range of metric types, including ratio, outcome, strategy, and kinetic metrics [84]. This permits a more extensive assessment of NMF by allowing practitioners to identify more than solely outcome measures (e.g., JH), which may offer limited utility for detecting neuromuscular fatigue [98], because changes in kinetic output can alter jump strategy alongside a maintenance in JH [84]. For example, monitoring JH alone might indicate a maintenance of NMF (i.e., minimal neuromuscular fatigue) following activity, which could be misleading if other kinetic and strategy metrics change concurrently [84]. Similarly, when assessing full-time professional English National League soccer players, Spencer et al. [73], reported no significant difference in CMJ JH from pre- (-90 min) to post- (+15 min) a competitive soccer match. However, significant (*p *< 0.05) reductions in body mass, jump momentum, and countermovement depth were seen. A maintenance of JH when body mass has decreased indicates a negative change in kinetic output, specifically, less net propulsive impulse (which is equal to jump momentum) has been produced overall.

Out of the 30 studies included within this review, only Spencer et al. [73] and Philipp et al. [75] monitored acute changes in body mass (derived from the CMJ test) from pre- to post- a physical stimulus, with both studies having been published since 2023. It may be prudent for practitioners to consider monitoring acute changes in body mass in addition to a combination of other categories such as ratio (e.g., RSI, mRSI), strategy (e.g., GCT, time-to take-off, countermovement depth), and kinetic (e.g., mean and peak propulsive force and power) metrics rather than assessing the outcome (e.g., JH) alone [84], to provide a better representation of changes in NMF over time [36,78,99–104]. This review identified a range of CMJ kinetic measures utilised in research monitoring acute changes in NMF, with some of the more frequently reported examples including peak (N = 7) and mean (N = 5) force, peak (N = 6) and mean (N = 4) power, and peak velocity (N = 3). This is a greater variety and frequency of metrics in comparison to findings of Guthrie et al. [12] who, despite showing commonality in the frequent use of peak power (N = 3) across studies evaluating longitudinal changes in physical preparedness, the only other kinetic measure reported was “relative power” which featured only once out of thirty-one studies included within the review [12]. The review by Guthrie et al. [12] also did not identify the use of any strategy metrics, whilst the current review identified a variety of them, including eccentric (N = 7) and concentric (N = 7) phase time, and total movement time (N = 4), which are important to consider when evaluating NMF using dynamic tasks given that the strategy of a VJ directly impacts its outcome (i.e., JH) [43].

A CMJ ratio metric identified within this review (N = 3) and the review of Guthrie et al. [12] (N = 2) was FT:CT. Despite metric popularity, in order for the chosen force plate metrics to be useful they must first be meaningful in that they are associated to independent measures of performance in the subject’s sport or occupation (e.g., strength, linear speed, and change of direction ability) [105]. Accordingly, CMJ metrics such as JH, peak force, and peak power have been related to independent “field-based” measures of strength and speed [105]. Given the importance of strength and speed for many athlete populations, it seems that a strong basis exists for including metrics such as JH, peak or mean force, and peak power, during CMJ testing [105]. Additionally, although specific metrics might be determined as meaningful in that they are strongly associated with independent physical performance measures, a metric’s reliability will ultimately determine its ability to detect acute changes in NMF [106]. Consequently, the test-retest reliability of these frequently reported ratio, outcome, strategy, and kinetic metrics should be determined before appropriate practical conclusions can be constructed. Whilst a variety of DJ metrics were identified in this review (N = 15), there was a lack of uniformity in metric selection with only peak force (N = 2) and contact time (N = 2) being reported more than once (Table 7). Because fast SSC tasks must be performed with a GCT of less than 250 ms to be considered truly “plyometric” [17], it seems reasonable that contact time would be a popular metric for articles utilising this test. To meet this requirement, fast SSC tests are required to be performed with a “stiff” strategy [43]. This typically results in greater peak forces than similar tests performed with a more complaint strategy (e.g., depth jumps) [43], which is also why it is understandable that peak force was a commonly reported DJ metric (Table 7).

Because of a focus on the standardisation of contact time in fast SSC tasks, and the frequent use of JH as an indicator of VJ performance [12], ratio metrics such as the RSI have been proposed in literature as a metric which indicates how “fast and high” a fast SSC test is performed [37]. If monitoring multiple metrics concurrently to determine changes in NMF, it might be common to find a scenario where one metric changes but another does not [105]. In this instance, it seems challenging to determine whether overall NMF has truly gotten better, worse, or not changed [105]. Bishop et al. [84] suggested that practitioners may wish to consider ‘linking metrics together’ (e.g., utilise ratio metrics) when interpreting data from VJ testing, as a way to utilise separate aspects of useful information concurrently [105]. It seems logical to assume that combining information about said metrics to form ratio metrics would streamline the monitoring process, but the individual components of any ratio metric also need to be considered to provide context to change. The use of RSI has been reported in research evaluating longitudinal developments in NMF [12], but featured only once in research monitoring acute changes in NMF by Merrigan et al. [56] (Table 7). Future research on monitoring acute changes in NMF should determine the utility of ratio metrics for this purpose. Furthermore, where a lack of research was identified utilising concentric-only (i.e., the SJ), unilateral, and isometric assessments, all associated metrics also only featured once (Tables 7 and 8). This gap in research is especially noticeable in the study within this review which reported the use of the “isometric posterior lower-limb muscle test” [58], where only peak force was the metric of interest (Table 9). This was also seen in the articles included in the review of Guthrie et al. [12], who reported the use of only peak force and RFD during the IMTP test, but this is understandable as these tests are typically employed to measure neuromuscular “strength” [107].

In recent years, there has been a growing interest into the concept of comparing the NMF of one limb to the other (i.e., evaluating inter-limb neuromuscular asymmetries) [108], likely due to the relation of greater inter-limb strength asymmetries (i.e., > 15%) to reduced VJ outcome and power output [109], poorer agility performance [110], heightened risk of anterior cruciate ligament (ACL) injury [111], and a suggested importance of utilising appropriate tests and metrics to monitor inter-limb changes in NMF during rehabilitation and return to play from ACL injury [110,112]. However, inter-limb asymmetries are not uncommon in athletes who will demonstrate a differential preference (i.e., subjectively preferred) and dominance (i.e., objectively greater NMF) of limb dependent on the specific sports task [113]. It has been discussed that a best practice method of determining limb dominance is yet to be proposed in literature [112], yet the preferred limb to execute a unilateral standing balance test and the primary leg to kick a soccer ball was reportedly the same in 66.7% and 85% of male and female healthy adults, respectively, in a study by van Melick et al. [112]. Of the four studies included in this review which utilised unilateral assessments [21,57,58,72], only Troester et al. [57] described a method of determining subjects’ preferred or dominant leg (Table 9). As suggested in a narrative review by Virgile and Bishop [113], future research should aim to clarify whether limb preference or dominance was considered in research investigating inter-limb asymmetries. It seems prudent to also recommended that the authors of future research describe the methods utilised to determine limb preference or dominance, and calculate inter-limb asymmetries, so that future comparisons of data reported in research can be performed accurately. Future research should also look to explore concentric-only, unilateral, and isometric assessments to discover their utility for monitoring acute changes in NMF. If performed with appropriate data collection and analysis methodologies, the information from future studies can be accumulated for meta-analyses to inform future practice.

### Data analysis procedures

This review has identified a lack of reporting of data analysis procedures across studies on monitoring acute changes in NMF, specifically, failing to provide calculations for CMJ (N = 62 out of 90), DJ (N = 9 out of 15), SJ (N = 3 out of 5), and unilateral test (N = 2 out of 13) metrics (Tables 5–8). Without the context of how metrics were calculated, data cannot be accurately compared across studies. For example, the calculation of CMJ JH varied most out of any metric reported across studies (N = 5), such as via TOV (TOV^2^/ 2 * g) [60], peak velocity [31,32,44], FT (via 1/2 g * [FT/ 2]^2^ [54], and, [g^-2^ * FT^2^]/ 8 [63]), and as the height of the COM at the apex minus at the point of take-off [51]. It is recommended, particularly when utilising force plates which apply forward dynamics procedures and follow guidelines of the impulse-momentum theorem to determine TOV [30], that JH should be determined via the TOV method [38]. For comparison, utilising peak velocity would inflate JH as this occurs prior to the instant of take-off (i.e., prior to plantar flexion). Thus, it is concerning that the TOV method was only utilised 3 times out of the 14 studies which reported CMJ JH (Table 6). Varying calculations were also identified for kinetic measures such as peak force (N = 2), namely as either the maximum force achieved throughout the entirety of the CMJ trial [31,32,44,55,65], or as the maximum force achieved during the propulsion phase [51,64]. This is a distinct difference, as the maximum force achieved during a CMJ can occur during the braking phase, prior to the initiation of the propulsion phase [76]. Thus, comparing “peak force” calculated in these separate ways would not be correct, where the latter would rather be best defined as “peak propulsive force” [76].

Peak concentric force was also reported (N = 4) and calculated as the maximal vertical force during the concentric phase in two separate studies [53,60]. Although this metric calculation better fits the metric description, and was consistent across the studies reporting it [53,60], this metric calculation matched that reported for peak force in the study by Oliver et al. [64] (i.e., the greatest force generated during the propulsion phase). Differing phase terminology creates an issue for data comparison (e.g., via meta-analysis) and for interpretation of information from studies for use in practice. In this review, varying phase terminology, such as braking (N = 15), eccentric (N = 13), propulsive (N = 10), and concentric (N = 19) was identified across all studies (Tables 5–8). Like the differences presented above, CMJ phase terminology such as eccentric phase time [31,32,44] and concentric phase time [31,32,44] have been utilised, and mean force has been calculated as the mean force generated during the concentric phase of the jump [31,32,44] and the mean force generated during the propulsion phase [64], in separate studies. Merrigan et al. [2] identified that the braking and propulsion phases of a CMJ test (as defined in HD Inc. proprietary software) have been incorrectly described as the eccentric and concentric phases, respectively, in separate commercially available force plate software, and the braking phase has been referred to as the eccentric phase in other published research [78,102,114]. This is potentially based on the assumption that the leg extensor muscles are actively lengthening (i.e., working eccentrically) to decelerate the body’s COM during this phase [76]. However, the collection and analysis of vertical force-time data using force plates only provides insight into the kinetics and kinematics of linear COM motion and cannot inform us as to what is occurring at the joint or muscle-tendon unit (MTU) level [76].

It is not only the leg extensors that contribute to the CMJ test, and it would be incorrect to assume that all lower-body MTUs are working eccentrically during the braking phase (e.g., the medial gastrocnemius may shorten during this phase [115]). These issues were also raised by Hahn [116] in a letter to the editor, who proposed the use of mechanical CMJ phase definitions such as unweighting, braking, and propulsion phases, determined via vertical COM velocity, in future research. However, Hahn [116] did not highlight the incorrect assumptions related to phase descriptions based on whether fascicles were actively lengthening (i.e., eccentric) or shortening (i.e., concentric) during these actions. Thus, as proposed in the work of McMahon et al. [76], the phases in VJ tasks which are calculated as from the instant of peak negative COM velocity until when COM velocity increases to zero (which coincides with the peak negative COM displacement), and from when a positive COM velocity is achieved until the instant of take-off, should be described as the “braking” and “propulsion” phases, respectively, and not the “eccentric” and “concentric” phases, respectively. It is also evident from the results of this review that not only have the same metric terminologies been utilised with differing metric calculations, but the same calculations have been prescribed to differing metric terminologies in research monitoring acute changes in NMF (Tables 5–8). In combination with the described differing use of phase terminology, this makes the comparison of study data extremely difficult and unlikely. Such a difference was identified for CMJ total movement time and take-off phase time, which were calculated as the time required to perform both the eccentric and concentric phase in separate studies (Table 6). This issue also extended to the calculation of power metrics such as CMJ peak power, where multiple calculations were reported (N = 4) across the 6 studies which utilised the metric (Table 6).

The greatest instantaneous power produced during the propulsion phase [51], peak force multiplied by peak velocity in the propulsive phase [55], the peak power achieved throughout the entirety of the CMJ trial [31,32,44], and peak force multiplied by peak velocity [65] were reported calculations of CMJ peak power across studies. This example highlights distinct differences between the determination of a peak value throughout the entirety of a trial versus specifically during the propulsive phase. For the remainder of test metrics identified in this study, only the DJ test demonstrated more than one metric calculation difference (Table 7). Specifically, contact time was calculated as the duration from contact (when forces were > 5 SDs above the one-second quite weighing phase average) to take-off (when forces were < 5 SDs of the quite weighing phase) [56], and the period between when force change by more than 10 N from resting body weight (initiation of movement), to when force was < 10 N (instant of take-off) [64], in separate studies. This example highlights a difference in phase identification, specifically in the determination of the instant of take-off and touchdown utilising standardised force thresholds (i.e., 10 N) [64] vs based on utilising 5 SDs of FT force [56] as recommended by McMahon et al. [76]. This is an issue as a difference in phase identification can affect the calculation of temporal aspects (e.g., contact time) and ratio metrics which include temporal measures within their calculations (e.g., RSI). Additionally, caution must be taken when determining the instant of touch-down and take-off utilising a specific force threshold as residual noise in the system can exceed 10 N dependent on the surface used, thus, understanding the phase thresholds utilised is important for the accurate determination of phase specific metrics when comparing data [76]. The authors refer readers to the work of McMahon et al. [76] for the accurate determination of CMJ phases.

## Conclusions

Practitioners must apply monitoring strategies to manage neuromuscular fatigue and physical preparedness with valid, reliable, and sensitive measures [12]. The results of this review give an overview of the previously used methodologies for monitoring acute changes in NMF using force plates and aimed to highlight issues and gaps that can be explored in future research. Major differences were identified across all aspects of studies methodologies, such as in subject demographics (e.g., sex, sport, and competitive level), data collection protocols (e.g., force plate hardware utilised, test and metric selection, verbal cues, and provision of information regarding testing surface, familiarisation and warm-up provided, the process of zeroing force plates between trials, and weighing of subjects during trials), and study design (e.g., reference physical activity investigated, time of season, testing timepoints, and TL determination). Additionally, the general lack of reporting and uniformity in metric definitions, metric calculations, and phase terminology across studies means an accurate comparison of results across studies (e.g., via meta-analysis) may not be possible, and any kind of generalized conclusions about the application of specific tests and metrics for monitoring acute changes in NMF using force plates would be premature at this time. With the recent growth in the utilisation of force plate measurements in real-world settings [80,117], the production of research centred on developing and promoting standardised testing procedures to determine capable tests and metrics for the acute monitoring of changes in NMF using force plates seems like a logical suggestion for future investigations.

Research is required to be employed with appropriate and standardised study designs across various sports populations, where research determining metrics’ sensitivity to change should be conducted in real-world environments where the information will be applied, for example, in team-sports (e.g., soccer) this could be applied around competitive matches (e.g., within 15 minutes pre- and post- an in-season competitive match), whilst reporting context of TL determination, if the intended data is to inform recovery processes. Additionally, it is important that studies report details of data collection procedures, such the surface used, warm-up and familiarisation protocols, process of zeroing force plates between trials, method of weighing participants during trials, and prescribed verbal cues to allow for replication and provide readers with confidence in the study’s results. The determination of the reliability and sensitivity to change of various popular tests (e.g., CMJ, DJ, etc.) and range of metric types, including ratio, outcome, strategy, and kinetic metrics [84] is required to develop a suitable and well-informed best practice methodology. Finally, it is non-negotiable that future research should be conducted with appropriate data analysis procedures (i.e., correct metric terminology, calculations, phase identifications, and phase terminology) and report these procedures clearly in their methods sections. An example of a validated approach is available via the HD Inc. force plate system’s proprietary software, as reported by Merrigan et al. [2]. These aspects will allow for the comparison of results in future research (e.g., via meta-analysis), for a better translation of knowledge into practice [87], and ultimately, allow the monitoring of acute changes in NMF to be adequately applied in practice.

## Supporting information

S1 Table Preferred Reporting Items for Systematic reviews and Meta-Analyses extension for Scoping Reviews (PRISMA-ScR) Checklist. (DOCX)
